# The role of ivermectin in the prevention and treatment of SARS-CoV-2 infection: a meta-analysis of randomized controlled trials

**DOI:** 10.1186/s12879-026-13195-9

**Published:** 2026-03-31

**Authors:** Xuanyu Wang, Jiahao Meng, Yinghui Li, Yuqing Xiang, Yumei Wu, Yilin Xiong, Pan Liu, Shuguang Gao

**Affiliations:** 1 Department of Orthopaedics, Xiangya Hospital, Central South University, Changsha, Hunan 410008 China; 2 Xiangya School of Medicine, Central South University, Changsha, Hunan 410008 China; 3 Key Laboratory of Aging-Related Bone and Joint Diseases Prevention and Treatment, Xiangya Hospital, Ministry of Education, Central South University, Changsha, China; 4 Central South University of Forestry and Technology, Changsha, Hunan 410004 China; 5 National Clinical Research Center for Geriatric Diseases (Xiangya Hospital), Central South University, Changsha, Hunan China; 6 Department of Sports Medicine, Xiangya Hospital, Central South University, Changsha, China

**Keywords:** COVID-19, Meta-analysis of randomized controlled trials, Prevention, Prognosis, Ivermectin

## Abstract

**Background:**

Ivermectin, as a potential drug for the treatment of Severe Acute Respiratory Syndrome Coronavirus 2 (SARS-CoV-2) infection, remains controversial regarding its efficacy and safety. This study aims to systematically evaluate the therapeutic and preventive effect of ivermectin in patients with SARS-CoV-2 infection.

**Methods:**

A comprehensive literature search was conducted on October 11, 2025, to include randomized controlled trials (RCTs) assessing ivermectin for the treatment of SARS-CoV-2 infection. The primary outcome measures were mortality rate and adverse event rate for hospitalized patients and outpatient patients, while secondary outcomes included hospitalization time and recovery time. Given the anticipated clinical and methodological heterogeneity across the included RCTs (e.g., variations in ivermectin dosage, study population characteristics, trial implementation time and SARS-CoV-2 variants), a random-effects model was used for the meta-analysis to obtain a robust synthesis of heterogeneous study results and reliable estimation of pooled effect sizes.

**Results:**

A total of 40 RCTs involving 23,243 participants were included. Among them, 4 studies evaluated the preventive effect of ivermectin on SARS-CoV-2 infection, and the other 36 studies evaluated the therapeutic effect of ivermectin in patients with SARS-CoV-2 infection. For prevention, there was no statistically significant difference between the ivermectin group and control group in SARS-CoV-2 infection rate [risk ratio (RR) 0.37; 95% Confidence Interval (CI) 0.12 to 1.20]. For treatment, all-cause mortality for both hospitalized patients (RR 0.94; 95%CI 0.74 to 1.20) and outpatients (RR 0.88; 95%CI 0.55 to 1.42) showed no statistically significant difference. Adverse events for hospitalized patients (RR 1.02; 95%CI 0.76 to 1.37) and outpatients (RR 0.96; 95%CI 0.82 to 1.13) also showed no statistically significant difference.

**Conclusions:**

This meta-analysis provides evidence that ivermectin does not statistically significantly reduce the risk of SARS-CoV-2 infection or improve clinical outcomes in patients with COVID-19. Further high-quality trials are needed to clarify the potential benefits of ivermectin.

**Clinical trial number:**

Not applicable.

**Supplementary Information:**

The online version contains supplementary material available at 10.1186/s12879-026-13195-9.

## Introduction

Coronavirus Disease 2019 (COVID-19), caused by Severe Acute Respiratory Syndrome Coronavirus 2 (SARS-CoV-2), has become a global public health emergency. As of 2025, the cumulative number of confirmed cases worldwide has exceeded 750 million, with over 6.8 million deaths [1]. The search for effective therapeutic methods remains a global research focus.

Ivermectin is an Food and Drug Administration (FDA) approved antiparasitic drug that has attracted attention in recent years due to its potential antiviral activity. In vitro studies have shown that ivermectin may reduce SARS-CoV-2 viral load by inhibiting the importin-α1 protein, which subsequently affects the nuclear transport of viral proteins [2, 3]. However, its basic binding mode and inhibitory mechanism remain unclear [4].

Despite positive results from in vitro studies, the efficacy of ivermectin in clinical settings remains controversial. Some meta-analyses have reported benefits of ivermectin in reducing mortality and hospitalization rates, while others have found no significant effects [5–13]. For example, one meta-analysis showed that ivermectin was associated with a reduction in death risk (RR 0.39, 95% CI 0.20–0.74) [10], whereas another study found no statistically significant effect of ivermectin on survival rate (RR 0.911, 95% CI 0.732–1.135) [11].

This systematic review and meta-analysis aims to provide an updated and comprehensive synthesis of the available evidence to evaluate the preventive and therapeutic effects of ivermectin for SARS-CoV-2 infection, thereby providing the latest evidence support for COVID-19 management and contributing to the evidence base for potential therapeutic and preventive approaches.

## Methods

This meta-analysis was performed following the guidelines outlined in the Preferred Reporting Items for Systematic Reviews and Meta-analysis (PRISMA) checklist [14] and all PRISMA checklist items were completed. The study protocol was registered on PROSPERO (CRD42024543441).

### Search strategy

A comprehensive systematic literature search was performed using PubMed, Cochrane Library, Embase, and Web of Science from the inception of databases up until October 11, 2025. The search was performed using terms included various combinations of “ivermectin”, “COVID-19”, “SARS-CoV-2”, “randomized controlled trial”, etc. Detailed search strategies can be found in the supplement.

### Inclusion and exclusion criteria

Studies that met all of the following criteria were included: (1) Randomized controlled trials evaluating ivermectin for the prevention or treatment of SARS-CoV-2 infection; (2) Reporting at least one pre-specified outcome measure; (3) Published in English.

Studies that met one of the following criteria were excluded: (1) Non-randomized controlled trials; (2) Duplicate published studies; (3) Studies with unavailable full text or incomplete data.

### Data extraction and quality assessment

A team of four reviewers, operating in two separate pairs, independently extracted data and assessed study quality. The extracted data included study characteristics and outcomes, such as author, publication year, region, number of participants, demographic characteristics of participants, treatment methods, rates of ICU admission and mechanical ventilation, all-cause mortality, lengths of hospital stay, the rate of negative COVID-19 tests and mean duration to viral clearance.

Meanwhile, the Risk of Bias 2 (RoB 2.0) tool was used to evaluate the methodological quality of the included studies (eFigure1 in supplement) [15]. Five potential domains of bias (Selection of the reported result, Measurement of the outcome, Missing outcome data, Deviations from intended interventions, Randomization process) was evaluated. The assessment labeled the risk as a low, unclear, or high risk of bias. Additionally, to assess the quality of evidence, we used the GRADE approach (eTable 1 in supplement) [16]. Disagreements were resolved through discussion. We calculated the kappa statistic to assess the consistency of data extraction and quality evaluation [17].

### Primary and secondary outcomes

For treatment group, the primary outcomes were all-cause mortality and adverse events for hospitalized patients and outpatient patients. The secondary outcomes included hospitalization time, negative COVID-19 test rate, viral clearance time, the need for mechanical ventilation, recovery time and ICU admission rate. The hospitalization time and recovery time deserve special attention due to their clinical significance.

For preventive group, the primary outcome was the rate of SARS-CoV-2 infection and the secondary outcome was adverse events.

### Statistical analysis

Continuous variables were presented as mean and standard deviation, and binary variables as event count and total count. Variables were combined using the Inverse Variance method, with an assumed correlation coefficient (r) of 0.5 [18]; this default value was selected in accordance with the Cochrane Handbook for Systematic Reviews of Interventions [19], the authoritative guidance for meta-analysis, which recommends *r* = 0.5 for calculating mean difference (MD) and standard deviation (SD) from change scores when study-specific correlation coefficients or individual patient-level data are unavailable. The detailed calculation methods are provided in the eTable 2 of supplementary materials [18].

Results were reported as MD for continuous outcomes and risk ratio (RR) for binary outcomes, each with a 95% confidence interval (CI). A random-effects model was adopted primarily due to potential inter-study heterogeneity; additionally, this model yields more robust results [19–21].

Inter-study heterogeneity was assessed using two statistics: I² (a measure of inter-study agreement) and τ² (tau squared, an absolute measure of heterogeneity). We reported I² in forest plots to evaluate heterogeneity, while also providing τ² to quantify absolute between-study variation. We recognized that τ², as a complementary measure, is particularly valuable for capturing details of inter-study differences in random-effects model analyses [19–21].

And sensitivity analysis was used to address heterogeneity via the leave-one-out method. Sensitivity analysis excluding high-risk bias studies (*n* = 8) was added to assess robustness of the findings. Publication bias was assessed using funnel plots, Egger’s test, and Begg’s test. All statistical analyses were performed using Review Manager 5.4.1 [19–21].

### Subgroup analysis

Subgroup analyses were only feasible for some outcomes. Specifically, we performed these analyses by three factors: ivermectin dosage (low, medium, high) and time-stratified subgroup analysis (2020–2022 vs. 2023–2025). Dosage subgroups were defined as: low dose (0.2–0.3 mg/kg/day), medium dose (0.3–0.4 mg/kg/day), and high dose (> 0.4 mg/kg/day), consistent with dosing ranges in included RCTs and prior COVID-19 ivermectin studies.

Given the wide time span of the included studies, the evolution of SARS-CoV-2 variants represents a critical, biologically meaningful source of between-study heterogeneity. Extensive mutations in the spike protein, altered cellular tropism, and modified replication kinetics of the Omicron variant and its sublineages (compared with the ancestral strain and pre-Omicron variants of concern such as Delta) can directly alter viral susceptibility to antiviral agents, including ivermectin [22]. The highly transmissible SARS-CoV-2 Omicron (B.1.1.529) variant was first reported in late 2021. While its BA.1 and BA.2 sublineages dominated the early global Omicron epidemic waves, the BA.4 and BA.5 sublineages replaced them to become the globally dominant strains in mid-2022, with numerous further descendant sublineages continuously evolving thereafter [23]. We therefore used December 26, 2022 (the date of the full lifting of quarantine measures in China, which also coincided with the complete shift of locally circulating strains in China to Omicron subvariants) as the pre-specified cutoff to stratify studies into two eras: 2020–2022 (pre-Omicron and early Omicron transition period) and 2023–2025 (full Omicron subvariant dominant period), to explore the impact of viral evolution on the efficacy of ivermectin.

## Results

### Study selection

**Fig. 1:**
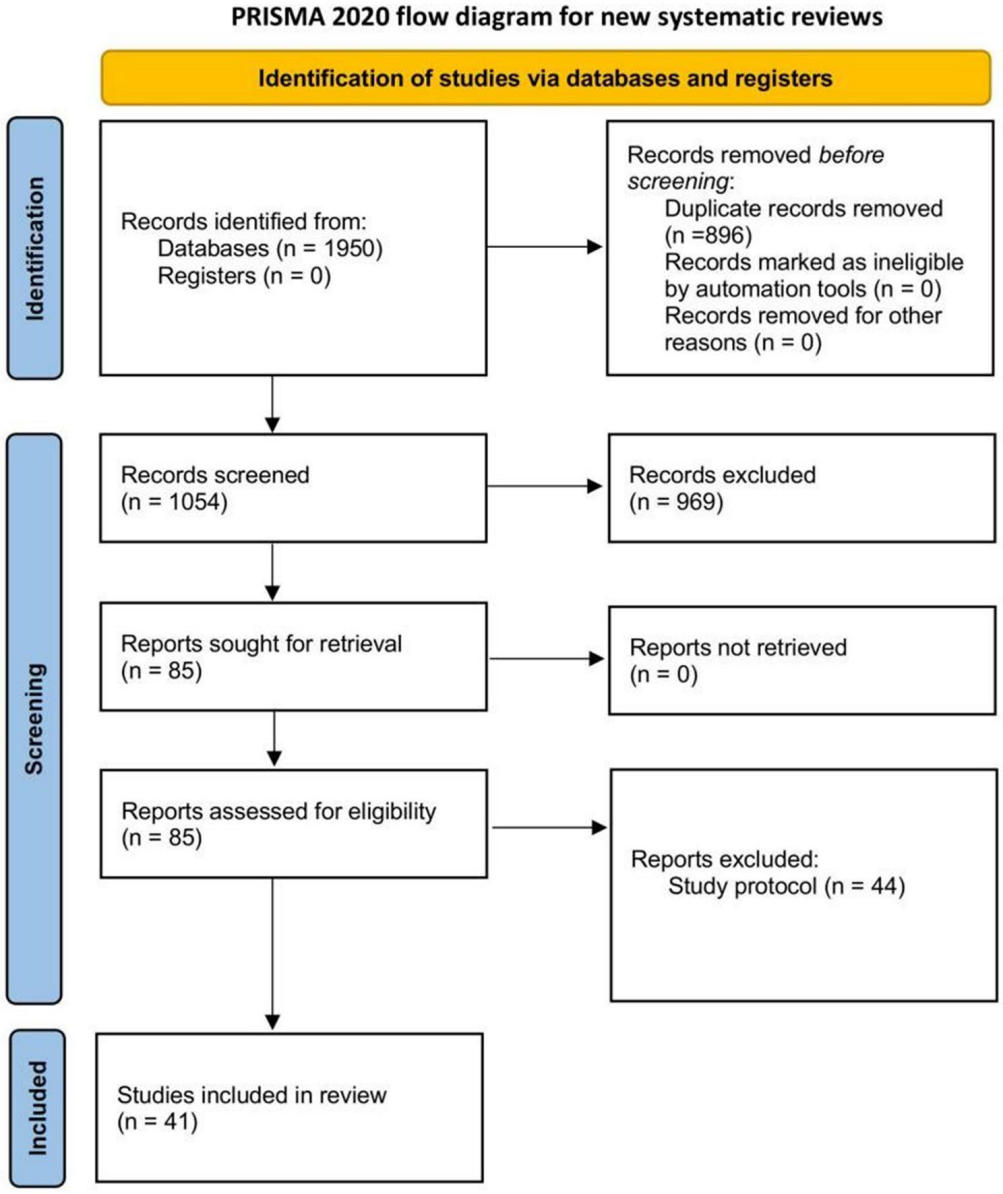
The literature search and selection process

The initial search yielded 1950 documents, and after screening, a total of 40 randomized controlled trials involving 23,243 participants were finally included. (eAppendix1 1–40) (Fig. 1). Among them, 4 studies [24–27] evaluated the preventive effect of ivermectin on SARS-CoV-2 infection, and the other 36 studies evaluated the therapeutic effect of ivermectin in patients with SARS-CoV-2 infection. In order to assess the inter-rater reliability of the study selection process, we calculated the kappa statistic, yielding a value of 0.82. A kappa value > 0.81 indicates very good inter-rater consistency. The details can be found in eFigure2 in the Supplementary Materials.

### Study characteristics

Regarding the preventive effects of ivermectin, a total of 1190 participants received ivermectin, with daily dosages ranging from 0.2 mg/kg to 0.6 mg/kg. As controls, 1109 participants received placebo or blank control. Regarding the therapeutic effects of ivermectin, a total of 10,068 participants received ivermectin, with daily dosages ranging from 0.2 mg/kg to 1.2 mg/kg. There were 10,876 participants who received control interventions (placebo or standard care alone). Participants in 22 studies were hospitalized patients, while participants in 14 studies were outpatients. With regard to dosage, 17 studies used low-dose ivermectin interventions (0.2–0.3 mg/kg/day), 11 studies used medium-dose ivermectin interventions (0.3–0.4 mg/kg/day), and 9 studies used high-dose ivermectin interventions (> 0.4 mg/kg/day). The complete standardized characteristics of all included RCTs, including detailed dosing regimens, study populations, intervention protocols and control measures, are summarized in eTable 3 in the supplementary materials.

### Quality assessment

With the using of RoB 2.0, the results showed that 30 studies had a low risk of bias, 8 studies had a high risk of bias, and the remaining 2 studies had a moderate risk of bias (eFigure1 in supplement). Notably, studies with a high risk of bias mainly had problems such as insufficient random sequence generation, inadequate allocation concealment, or imperfect blinding.

### Primary outcomes

**Fig. 2:**
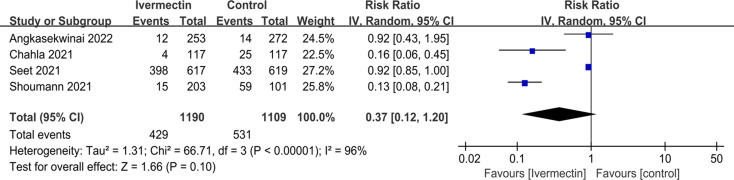
Preventive effects of ivermectin on SARS-CoV-2 infection

For the preventive group, there was no statistically significant difference between the ivermectin group and control group in SARS-CoV-2 infection rate (RR 0.37; 95%CI 0.12 to 1.20) (Fig. 2).

**Fig. 3:**
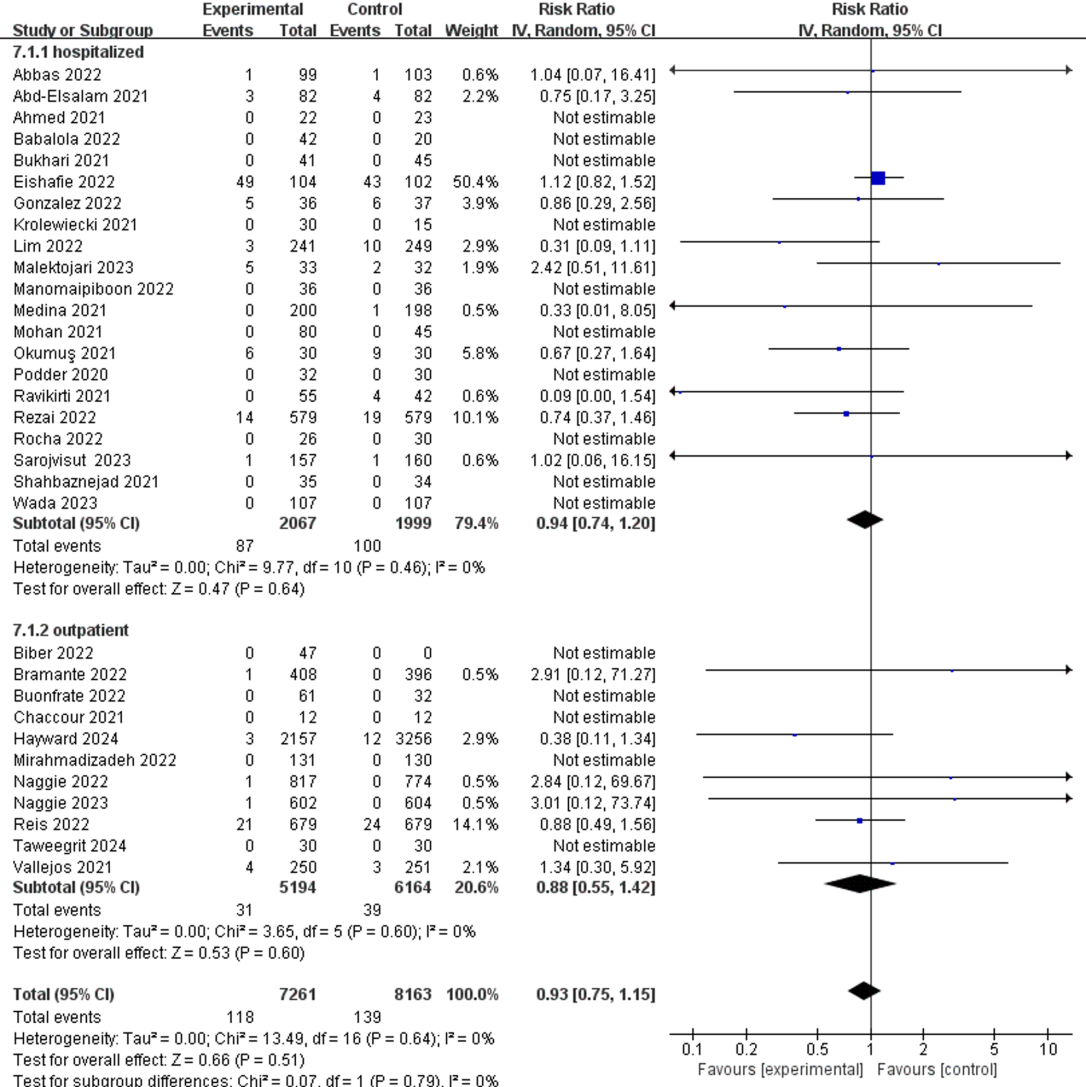
All-cause mortality of ivermectin and control group on SARS-CoV-2 infection

**Fig. 4:**
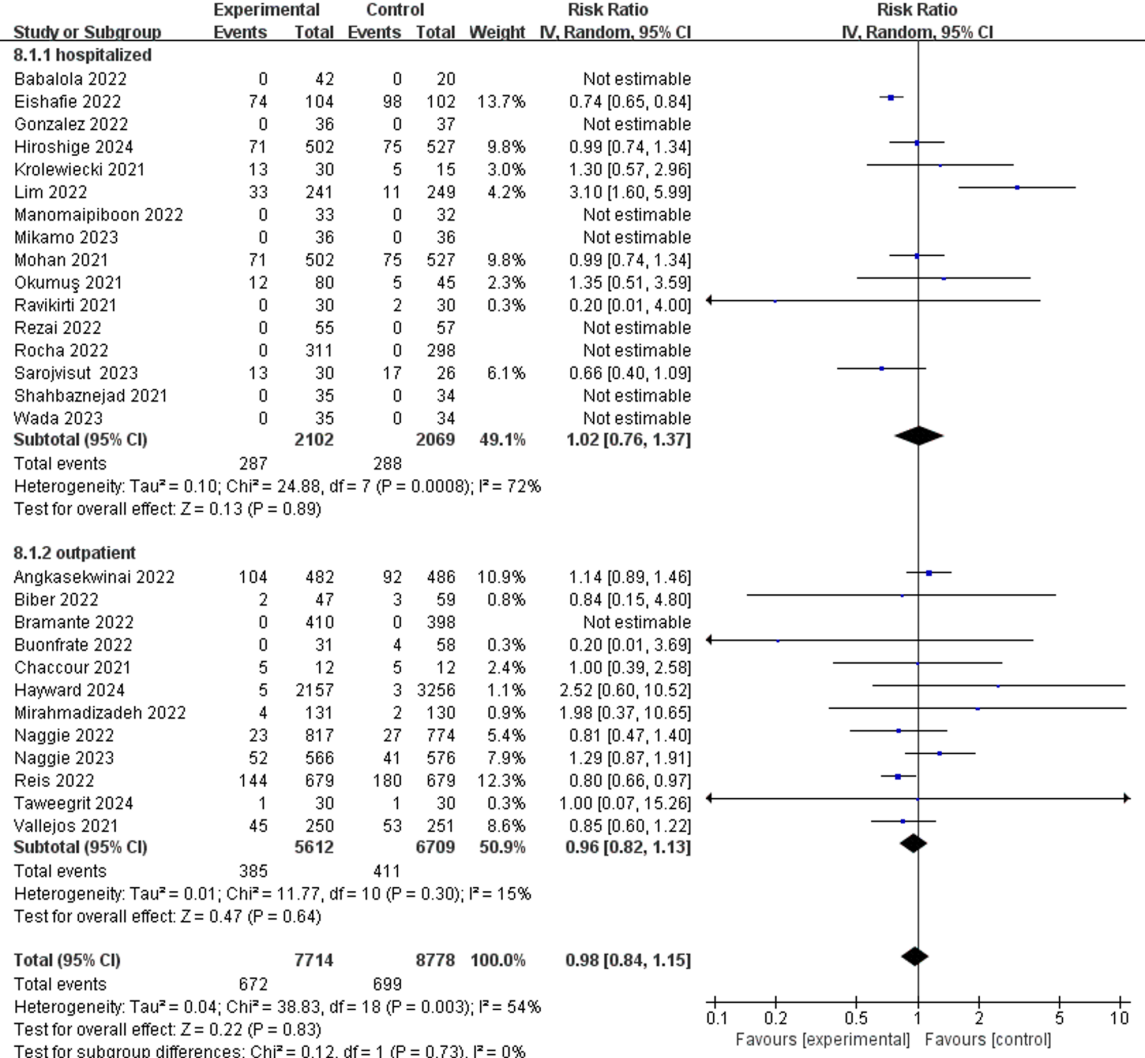
Adverse events of ivermectin and control group on SARS-CoV-2 infection

For treatment group, all-cause mortality for both hospitalized patients (RR 0.94; 95%CI 0.74 to 1.20) and outpatients (RR 0.88; 95%CI 0.55 to 1.42) (Fig. 3) showed no statistically significant difference. Adverse events for hospitalized patients (RR 1.02; 95%CI 0.76 to 1.37) and outpatients (RR 0.96; 95%CI 0.82 to 1.13) (Fig. 4) also showed no statistically significant difference.

### Secondary outcomes

For the treatment group, the length of hospital stay (MD -0.05; 95%CI -0.59 to 0.49) (eFigure3 in supplement), the rate of negative COVID-19 tests (RR 0.91; 95%CI 0.81 to 1.03) (eFigure4 in supplement), the mean duration to viral clearance (MD -1.21; 95%CI -4.35 to 1.92) (eFigure5 in supplement), rates of ICU admission (RR 1.12; 95%CI 0.64 to 1.96) (eFigure6 in supplement), recovery time (MD -0.31; 95%CI -2.41 to 1.79) (eFigure7 in supplement) and mechanical ventilation (RR 0.73; 95%CI 0.51 to 1.04) (eFigure8 in supplement), had no statistically significant difference.

For the preventive group, the pooled results showed that there was no statistically significant difference in adverse events (RR 1.08; 95%CI 0.89 to 1.30) (eFigure9 in supplement).

### Subgroup analysis

**Fig. 5:**
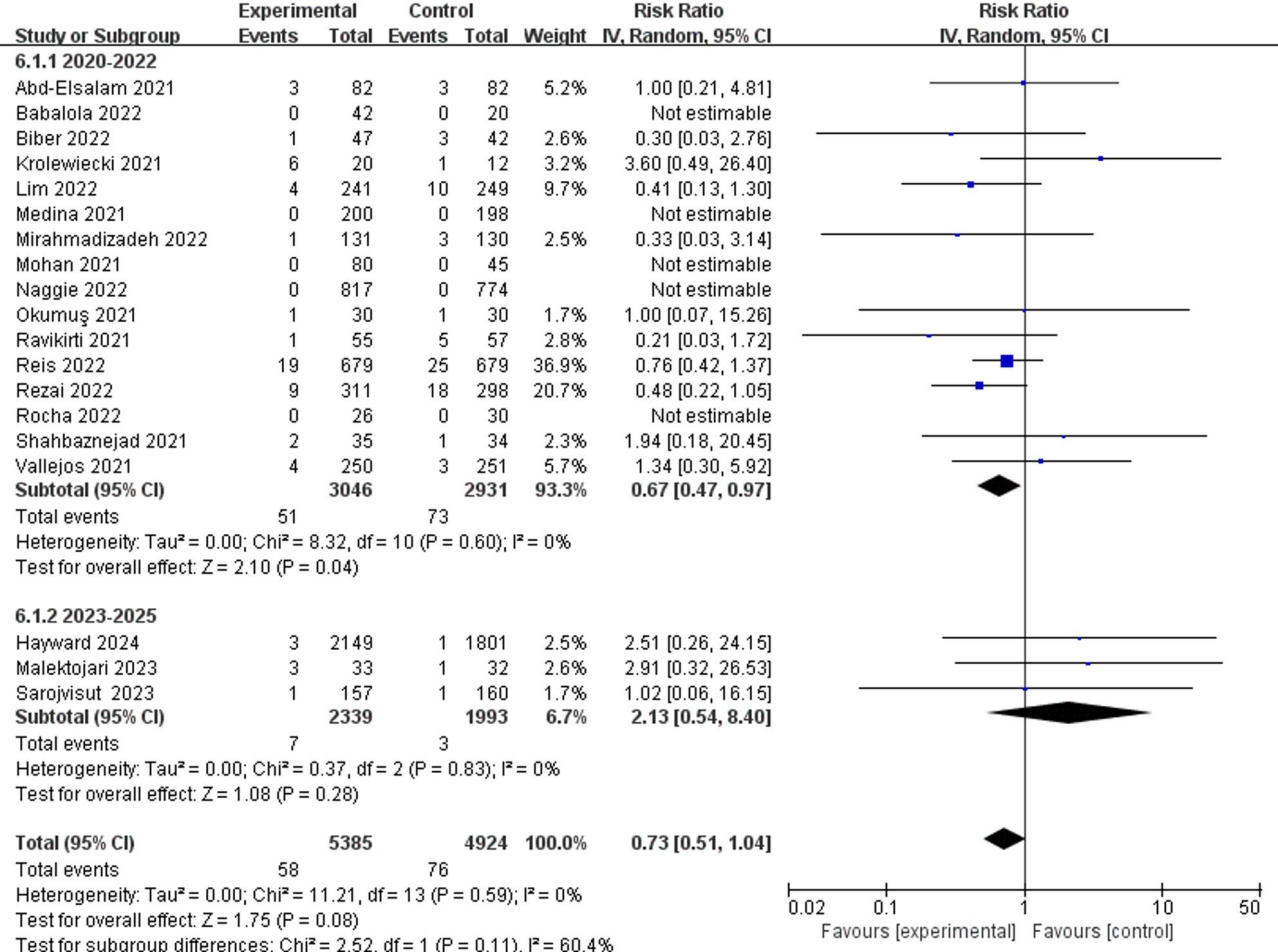
Time-stratified subgroup analysis of ivermectin’s effect on the need for mechanical ventilation

Subgroup analysis by dose demonstrated no statistically significant effect (eFigure10 in supplement). Meanwhile, the time-stratified subgroup analysis’s pooled results indicated that ivermectin had a statistically significant effect on reducing mechanical ventilation in studies conducted between 2020 and 2022 (RR 0.67; 95%CI 0.47 to 0.97), whereas no statistically significant effect was observed in those from 2023 onwards (RR 2.13; 95%CI 0.54 to 8.40) (Fig. 5).

Notably, while this 2023–2025 time-stratified subgroup analysis is novel in capturing data from the full Omicron subvariant-dominant era (a period largely unaddressed in prior meta-analyses), it includes only 5 studies, with wide 95% confidence intervals indicating limited statistical power and low precision of the pooled effect estimate. It is worth mentioning that the pre-2022 signal of reduced mechanical ventilation should be interpreted with caution due to concurrent improvements in standard care, which may have confounded treatment effect estimates. In either time-stratified subgroup, the pooled results consistently showed that ivermectin had no statistically significant effect on reducing all-cause mortality (eFigure11 in supplement) and adverse event analyses (eFigure12 in supplement).

### Sensitivity analysis

Sensitivity analysis excluding high-risk bias studies: All-cause mortality (RR 0.93; 95% CI 0.74–1.18; I²=0%), consistent with full analysis. And the results including or excluding grey literature are also robust.

### Publication bias

We plotted a funnel plot for outcomes with > 10 included studies, and the results showed slight asymmetry (eFigure13-15 in supplement). We performed Egger’s and Begg’s statistical tests [28] for all outcomes and all outcomes showed *p* > 0.05. However, this result needs to be interpreted with caution due to the limited sample size.

## Discussion

This systematic review and meta-analysis of 40 randomized controlled trials evaluated the prophylactic and therapeutic efficacy of ivermectin against SARS-CoV-2 infection. A core finding was that ivermectin exerted no statistically significant effects on mortality and the incidence of adverse events in the overall analysis. And there’s no statistically significant difference between the length of hospital stay (MD -0.05; 95%CI -0.61 to 0.50) and recovery time (MD -0.46; 95%CI -2.94 to 2.01).

Several potential explanations may underlie the observed lack of efficacy. First, the polarity-nonpolarity characteristics of ivermectin—a lipophilic macrocyclic lactone —limit its bioavailability and tissue distribution. Its high lipophilicity drives preferential accumulation in hepatic and adipose tissues, while its poor aqueous solubility and high plasma protein binding rate result in extremely low free drug concentrations available for penetration into respiratory epithelial cells, the primary site of SARS-CoV-2 replication. This leads to insufficient pharmacological resources at the biological target, may rendering antiviral activity unattainable in vivo [29].

Second, drug metabolism and pharmacokinetic constraints further compromise efficacy. Ivermectin is primarily metabolized by the cytochrome P450 3A4 enzyme system (with minor contributions from CYP3A5 and 2C9) through C-hydroxylation and O-demethylation, resulting in the identification of 13 metabolites. When ivermectin is co-administered with P450 3A4 inhibitors/inducers or transporter substrate drugs, or in the presence of genetic polymorphisms of enzymes/transporters, drug-drug interactions may occur. Additionally, high-dose administration can induce toxicity. The in vitro effective concentration range (2–10 µM) is far exceeding the safe human dose, which is liable to trigger severe adverse reactions such as neurotoxicity [30].

Third, discrepancies between in vitro and in vivo effects reveal non-specific pharmacological artifacts. While ivermectin inhibits SARS-CoV-2 in Vero E6 cells, this activity is not reproducible in human primary bronchial epithelial cells—even at concentrations up to 10 mM [31, 32]. Mechanistic studies suggest in vitro activity stems from non-specific membrane perturbation (e.g., lipid bilayer disruption) and interference with assay readouts (e.g., aggregation-induced quenching of singlet oxygen in AlphaScreen), rather than specific antiviral targeting [33]. This explains why preclinical promise has not translated to clinical benefit, a critical point supported by recent mechanistic studies.

Our findings also address inconsistencies with best practices for SARS-CoV-2 prevention and treatment. Current guidelines from the WHO, IDSA, and FDA do not recommend ivermectin for routine use outside clinical trials, citing low-certainty evidence (GRADE: very low) and failure to meet core therapeutic criteria. Specifically, ivermectin violates best practices in three key areas: insufficient target-site drug concentrations (contradicting pharmacotherapeutic principles), lack of consistent efficacy across high-quality RCTs (undermining evidence-based practice), and potential for drug-drug interactions that increase safety risks.

### Comparison with existing literature and study significance

When contextualized within existing literature, our results align with some recent systematic reviews and meta-analyses [7, 9, 11, 12, 34] that both reported non-significant effects on mortality in hospitalized patients, consistent with our findings. And compared with recent meta-analyses on ivermectin and COVID-19 [9, 11–13, 35, 36], our study differs in inclusion criteria, sample size, outcome focus, and analytical rigor, effectively addressing unmet evidence gaps.

In terms of inclusion criteria and sample size, unlike Song’s research [13] with 33 studies and 10,489 participants and Satyam’s research [12] with 23 RCTs, 12,345 participant, who lacked the latest literature, we included newly published studies [37–39]. As to prevention effects, Zhou’s research [35] with 8 ivermectin studies, 3,120 participants focused on healthy adults’ prevention, data up to Sep 2022, while our analysis extended data collection to 2025, capturing efficacy against post-2022 Omicron subvariants via a novel time-stratified analysis. However, this 2023–2025 time-stratified subgroup analysis includes only 5 recent studies, which limits the strength of any conclusions regarding ivermectin’s efficacy against currently circulating SARS-CoV-2 variants. Maddukuri’s research [9] with 19 RCTs with 1,111 participants also failed to include 2023–2025 data. Sai’s research [11], while including 33 studies and 15,376 participants up to April 2024, focused primarily on therapeutic outcomes (mortality, mechanical ventilation) without systematic evaluation of preventive efficacy or time-stratified analysis.

Beyond updated trial data, our study has key methodological strengths that improve the rigor and clinical relevance of our findings compared with prior reviews. First, we implemented comprehensive quality control via full RoB 2.0 bias assessment for all 40 RCTs, a pre-specified sensitivity analysis excluding high-risk studies, and systematic GRADE evidence rating for all outcomes. Second, we conducted a pre-specified dose-stratified subgroup analysis to explore the impact of different ivermectin dosing regimens, an analysis omitted in most prior meta-analyses. Third, our time-stratified analysis identified divergent mechanical ventilation outcomes between pre-2022 and post-2023 trials, and explained this discrepancy via the confounding effect of evolving COVID-19 standard of care. Finally, our literature search extended to October 2025, filling a critical evidence gap by evaluating ivermectin’s efficacy against Omicron subvariants, a period largely unaddressed in prior reviews.

Regarding outcomes, contrary to Song’s research [13] which claimed ivermectin reduced mechanical ventilation and adverse events, and Andrew’s research [36] which found that large reductions in COVID-19 deaths are possible using ivermectin. Using ivermectin early in the clinical course may reduce numbers progressing to severe disease, our results showed no such effect. Notably, we conducted a unique time-stratified subgroup analysis (2020–2022 vs. 2023–2025), revealing that ivermectin reduced mechanical ventilation pre-2022 (RR 0.67, 95% CI 0.47–0.97) but not post-2023 (RR 2.13, 95% CI 0.54–8.40)—an analysis absent in prior studies. However, this isolated subgroup finding was not accompanied by any improvement in the core clinical endpoint of all-cause mortality, nor by benefits in other key clinical outcomes, and thus does not alter the overall conclusion that ivermectin has no clinically meaningful therapeutic effect. While Zhou et al. reported high heterogeneity (I²=96%) in preventive efficacy analysis without systematic exploration of its sources, our strict inclusion of only RCTs and standardized RoB 2.0 bias assessment helped reduce the risk of methodological heterogeneity from non-randomized study designs; meanwhile, we conducted pre-specified dose-stratified and time-stratified subgroup analyses to explore the potential sources of the remaining substantial heterogeneity in several outcomes. Additionally, although Maddukuri et al. and Sai et al. concluded no significant effect of ivermectin on mortality or hospitalization, they neglected detailed preventive efficacy assessment and dose-related differences, which we addressed via dosage-stratified subgroup analyses.

In summary, the necessity of our review lies in resolving three key limitations of prior work. First, it fills the evidence gap for 2023–2025, a period of dominant Omicron subvariants that previous studies did not cover. Second, it clarifies heterogeneity by stratifying by time and dosage and focusing on RCTs to addressing the high variability. Third, it summarizes the efficacy and safety of ivermectin application for prevention and treatment based on existing evidence.

Limitations.

Despite the comprehensive nature of this meta-analysis, several limitations should be acknowledged. First, the number of studies evaluating ivermectin for prevention of SARS-CoV-2 infection was relatively small (4 studies), leading to wide confidence intervals around the effect estimates and limited statistical power to detect small but potentially clinically meaningful effects. This small sample size also contributed to the high heterogeneity observed in the prevention analysis (I²=96%), which may reflect differences in study populations, intervention protocols, and exposure risks across these studies.

Second, the majority of included studies were conducted during the early phases of the COVID-19 pandemic (2020–2022), before the emergence of more recent SARS-CoV-2 variants such as Omicron subvariants. Our time-stratified analysis suggested that the potential benefit of ivermectin on mechanical ventilation observed in earlier studies was not present in more recent research (2023–2025). However, this 2023–2025 time-stratified subgroup analysis, while novel in addressing a critical evidence gap for recent circulating variants, includes only 5 studies. This small sample size results in severely limited statistical power, wide and imprecise confidence intervals for effect estimates, and an inability to detect small but potentially clinically meaningful treatment effects. Thus, no definitive conclusions can be drawn about ivermectin’s efficacy against recent Omicron subvariants, and this key limitation restricts the generalizability of our findings to the post-2022 pandemic period.

Third, despite our rigorous study selection and quality assessment, residual bias may remain due to limitations in individual study designs. Most included studies had low to moderate bias risk, but some had deficiencies in blinding and allocation concealment, which may compromise result validity and require analysis in conjunction with study design and outcome measures. Blinding is central to controlling subjective and objective biases. Due to intervention characteristics or resource constraints, some included studies adopted single-blind or non-blinded designs, easily causing observer and participant biases that distort the true presentation of intervention effects. Inadequate allocation concealment tends to induce selection bias. Some studies failed to specify methods or used simple randomization without sealed concealment, possibly undermining baseline balance between groups and falsely overestimating intervention efficacy. Nonetheless, these methodological limitations—despite not changing core conclusions via random-effects model—compromised internal validity.

Fourth, substantial statistical heterogeneity was observed across studies for most outcomes, particularly for adverse events (I²=73%). This heterogeneity may be attributed to differences in ivermectin dosing regimens (ranging from single doses to multiple days), timing of administration (pre-exposure prophylaxis vs. early treatment vs. late-stage disease), study populations (healthy adults vs. high-risk patients), and comparator interventions. While we conducted subgroup analyses to explore potential sources of heterogeneity, the limited number of studies in some subgroups prevented a comprehensive assessment, such as evaluating the impact of ivermectin in specific patient populations such as immunocompromised individuals or exploring potential interactions between ivermectin and other medications. Similarly, we could not comprehensively assess the impact of different dosing regimens or durations of treatment on outcomes, which may be important for optimizing clinical use.

### Future research directions

Despite the limitations of ivermectin in antiviral treatment, research on ivermectin in the field of antiviral therapy, particularly in resource-limited settings, still holds significant scientific value. By gaining a deeper understanding of its pharmacokinetic limitations and in vitro-in vivo conversion barriers, we can provide valuable experience for future drug development. In particular, the case of ivermectin serves as a reminder that when translating in vitro antiviral activity into clinical treatment, it is essential to fully consider the physicochemical properties, metabolic characteristics, and target tissue distribution of the drug, to avoid overestimating the clinical relevance of in vitro experimental results.

Future research should focus on optimizing drug administration routes, modifying chemical structures, developing metabolic inhibition strategies, and exploring drug delivery systems to overcome the limitations of ivermectin in antiviral treatment. Additionally, research on the impact of SARS-CoV-2 infection on the expression of drug-metabolizing enzymes should be intensified to provide a scientific basis for personalized medication.

## Conclusion

This meta-analysis provides evidence that ivermectin does not statistically significantly reduce the risk of SARS-CoV-2 infection or improve clinical outcomes (including all-cause mortality, hospitalization duration, and recovery time) in patients with COVID-19. Furthermore, this meta-analysis found no reliable statistical evidence that ivermectin either increases or decreases the rate of adverse events compared to a control group. Further high-quality, large-sample, prospectively designed randomized controlled trials are needed to clarify the potential clinical benefits and safety profile of ivermectin for SARS-CoV-2 infection.

## Supplementary Information

Below is the link to the electronic supplementary material.

Supplementary Material 1

## Data Availability

Data included in article/supplementary material/referenced in article.

## Abbreviations

RR: Risk ratio
RCT: Randomized controlled trial
PRISMA: Preferred Reporting Items for Systematic Reviews and Meta-analysis
CI: Confidence Interval
MD: Mean difference
