# Supplementary material for: The role of ivermectin in the prevention and treatment of SARS-CoV-2 infection: a meta-analysis of randomized controlled trials
Source: BMC Infect Dis. 2026 Mar 31;26:1035. doi: 10.1186/s12879-026-13195-9 (PMC13214460; doi:10.1186/s12879-026-13195-9)
Supplement: Supplementary file 1 — Supplementary Material 1 [file 12879_2026_13195_MOESM1_ESM.docx]

**Supplemental Online Content**

Search strategy P2-4

eFigure1.Risk of bias of each included study by ROB2 P5

eFigure2.Kappa of ivermectin for Covid-19 P6

eFigure3.Ivermectin on the length of hospital stay P7

eFigure4.Rate of negative COVID-19 tests P8

eFigure5.Mean duration to viral clearance P9

eFigure6.Rates of ICU admission P10

eFigure7.Recovery time P11

eFigure8.Rate of mechanical ventilation P12

eFigure9.preventive group with adverse events P13

eFigure10.Subgroup analysis on patients with low dosage,medium dosage and high dosage P14

eFigure11.time-stratified subgroup on reducing all-cause mortality P15

eFigure12.time-stratified subgroup on reducing adverse events P16

eFigure13.funnel plot of all-cause morality P17

eFigure14.funnel plot of adverse events P18

eFigure15.funnel plot of mechanical ventilation P19

eTable 1.Grading of ivermectin for Covid-19 P20-22

eTable 2.specific calculation methodology of MD and SD P23-24

eTable 3.Characteristics of included RCTs P25-29

eAppendix 1.Reference list of included studies P30-34

PubMed(315)

1.“COVID-19”[Mesh]OR"COVID-19"[tiab]OR“COVID 19”[tiab]OR“2019-nCoV Infection”[tiab]OR“2019 nCoV Infection”[tiab]OR“2019-nCoV Infections”[tiab]OR“Infection,2019-nCoV”[tiab]OR“SARS-CoV-2 Infection”[tiab]OR“Infection,SARS-CoV-2”[tiab]OR“SARS CoV 2 Infection”[tiab]OR“SARS-CoV-2 Infections”[tiab]OR“2019 Novel Coronavirus Disease”[tiab]OR“2019 Novel Coronavirus Infection”[tiab]OR“COVID-19 Virus Infection”[tiab]OR“COVID 19 Virus Infection”[tiab]OR“COVID-19 Virus Infections”[tiab]OR“Infection,COVID-19 Virus”[tiab]OR“Virus Infection,COVID-19”[tiab]OR“COVID19”[tiab]OR“Coronavirus Disease 2019”[tiab]OR“Disease 2019,Coronavirus”[tiab]OR“Coronavirus Disease-19”[tiab]OR“Coronavirus Disease 19”[tiab]OR“Severe Acute Respiratory Syndrome Coronavirus 2 Infection”[tiab]OR“COVID-19 Virus Disease”[tiab]OR“COVID 19 Virus Disease”[tiab]OR“COVID-19 Virus Diseases”[tiab]OR“Disease,COVID-19 Virus”[tiab]OR“Virus Disease,COVID-19”[tiab]OR“SARS Coronavirus 2 Infection”[tiab]OR“2019-nCoV Disease”[tiab]OR“2019 nCoV Disease”[tiab]OR“2019-nCoV Diseases”[tiab]OR“Disease,2019-nCoV”[tiab]OR“COVID-19 Pandemic”[tiab]OR“COVID 19 Pandemic”[tiab]OR“Pandemic,COVID-19”[tiab]OR“COVID-19 Pandemics”[tiab]

2."ivermectin"[MeSH Terms]OR"ivermectin"[All Fields]OR"ivermectine"[All Fields]OR"ivermectin s"[All Fields]OR"ivermectins"[All Fields]OR("ivermectin"[MeSH Terms]OR"ivermectin"[All Fields]OR"ivermectine"[All Fields]OR"ivermectin s"[All Fields]OR"ivermectins"[All Fields])OR(("ivermectin"[MeSH Terms]OR"ivermectin"[All Fields]OR"ivermectine"[All Fields]OR"ivermectin s"[All Fields]OR"ivermectins"[All Fields])AND"s"[All Fields])OR("ivermectin"[MeSH Terms]OR"ivermectin"[All Fields]OR"ivermectine"[All Fields]OR"ivermectin s"[All Fields]OR"ivermectins"[All Fields])OR("ivermectin"[MeSH Terms]OR"ivermectin"[All Fields]OR"ivermectine"[All Fields]OR"stromectol"[All Fields]OR"ivermectin s"[All Fields]OR"ivermectins"[All Fields])OR("ivermectin"[MeSH Terms]OR"ivermectin"[All Fields]OR"mectizan"[All Fields])OR("ivermectin"[MeSH Terms]OR"ivermectin"[All Fields]OR"mk 933"[All Fields])OR("ivermectin"[MeSH Terms]OR"ivermectin"[All Fields]OR"mk 933"[All Fields])OR("ivermectin"[MeSH Terms]OR"ivermectin"[All Fields]OR"mk933"[All Fields])OR("ivermectin"[MeSH Terms]OR"ivermectin"[All Fields]OR"eqvalan"[All Fields])OR("ivermectin"[MeSH Terms]OR"ivermectin"[All Fields]OR"ivermectine"[All Fields]OR"ivomec"[All Fields]OR"ivermectin s"[All Fields]OR"ivermectins"[All Fields])

3.((compar*[tiab])OR((singl*[tiab]or doubl*[tiab]or tripl*[tiab])and(mask*[tiab]or blind*[tiab])))OR(random*[tiab]or placebo[tiab]or controlled[tiab]or trial*[tiab])

4.#1 And#2 And#3

Cochrane Library(224)

1. MeSH descriptor:[COVID-19]explode all trees
2. (COVID-19 OR COVID 19 OR 2019 nCoV Infection OR SARS-CoV-2 Infection OR Infection,SARS-CoV-2 OR SARS CoV 2 Infection OR SARS-CoV-2 Infections OR 2019 Novel Coronavirus Disease OR 2019 Novel Coronavirus Infection OR COVID-19 Virus Infection OR COVID 19 Virus Infection OR COVID-19 Virus Infections OR Infection,COVID-19 Virus OR Virus Infection,COVID-19 OR COVID19 OR Coronavirus Disease 2019 OR Disease 2019,Coronavirus OR Coronavirus Disease-19 OR Coronavirus Disease 19 OR Severe Acute Respiratory Syndrome Coronavirus 2 Infection OR COVID-19 Virus Disease OR COVID 19 Virus Disease OR COVID-19 Virus Diseases OR Disease,COVID-19 Virus OR Virus Disease,COVID-19 OR SARS Coronavirus 2 Infection OR 2019 nCoV Disease OR COVID-19 Pandemic OR COVID 19 Pandemic OR Pandemic,COVID-19 OR COVID-19 Pandemics):ti,ab
3. #1 OR#2
4. MeSH descriptor:[Ivermectin]explode all trees
5. (ivermectin):ti,ab,kw OR(ivermectine):ti,ab,kw OR(ivermectin s):ti,ab,kw OR(ivermectins):ti,ab,kw OR(Stromectol):ti,ab,kw OR(Mectizan):ti,ab,kw OR(MK-933):ti,ab,kw OR(MK 933):ti,ab,kw OR(Eqvalan):ti,ab,kw OR("Ivomec"):ti,ab,kw
6. #4 OR#5
7. ((compar*)OR((singl*or doubl*or tripl*)and(mask*or blind*)))OR(random*or placebo or controlled or trial*):ti,ab
8. #3 AND#6 AND#7

Embase(805)

1. 'coronavirus disease 2019'/exp
2. ((Covid-19)OR(Covid 19)OR(2019-nCoV Infection)OR(SARS-CoV-2 Infections)):ti,ab
3. #1 OR#2
4. invermectin OR'ivermectin'/exp OR'ivermectin'
5. (ivermectin:ti,ab,kw OR ivermectine:ti,ab,kw OR(ivermectin:ti,ab,kw AND s:ti,ab,kw)OR ivermectins:ti,ab,kw OR stromectol:ti,ab,kw OR mectizan:ti,ab,kw OR'mk 933':ti,ab,kw OR(mk:ti,ab,kw AND 933:ti,ab,kw)OR eqvalan:ti,ab,kw OR'ivomec':ti,ab,kw):ti,ab
6. #4 OR#5
7. #3 AND#6
8. ((compar*)OR((singl*or doubl*or tripl*)and(mask*or blind*)))OR(random*or placebo or controlled or trial*):ti,ab
9. #7 AND#8

Web of Science(671)

1. TS=(COVID-19 OR COVID 19 OR 2019-nCoV Infection OR 2019 nCoV Infection OR 2019-nCoV Infections OR Infection,2019-nCoV OR SARS-CoV-2 Infection OR Infection,SARS-CoV-2 OR SARS CoV 2 Infection OR SARS-CoV-2 Infections OR 2019 Novel Coronavirus Disease OR 2019 Novel Coronavirus Infection OR COVID-19 Virus Infection OR COVID 19 Virus Infection OR COVID-19 Virus Infections OR Infection,COVID-19 Virus OR Virus Infection,COVID-19 OR COVID19 OR Coronavirus Disease 2019 OR Disease 2019,Coronavirus OR Coronavirus Disease-19 OR Coronavirus Disease 19 OR Severe Acute Respiratory Syndrome Coronavirus 2 Infection OR COVID-19 Virus Disease OR COVID 19 Virus Disease OR COVID-19 Virus Diseases OR Disease,COVID-19 Virus OR Virus Disease,COVID-19 OR SARS Coronavirus 2 Infection OR 2019-nCoV Disease OR 2019 nCoV Disease OR 2019-nCoV Diseases OR Disease,2019-nCoV OR COVID-19 Pandemic OR COVID 19 Pandemic OR Pandemic,COVID-19 OR COVID-19 Pandemics)

2.TS=(ivermectin*OR stromectol*OR mectizan*OR"MK 933"OR MK933 OR eqvalan*OR soolantra*OR sklice*OR stromectal*OR ivomec*)OR AB=(ivermectin*OR stromectol*OR mectizan*OR"MK 933"OR MK933 OR eqvalan*OR soolantra*OR sklice*OR stromectal*OR ivomec*)

3.#1 AND#2

4.TS=(((compar*)OR((singl*or doubl*or tripl*)and(mask*or blind*)))OR(random*or placebo or controlled or trial*))

5.#3 AND#4


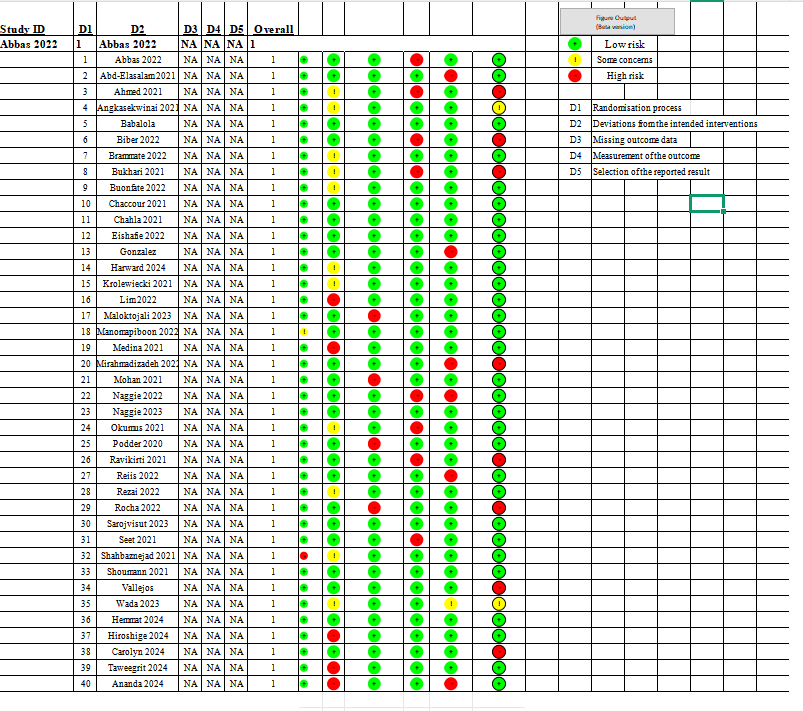


eFigure1.Risk of bias of each included study by ROB2

author1=c(1,1,3,2,1,2,3,1,1,2,3,3,2,1,1,2,3,1,1,3,1,3,2,3,1)

author2=c(1,1,3,2,1,2,3,1,1,2,2,3,2,1,1,2,2,1,1,2,1,3,2,3,1)

The Kappa value can be calculated using the following formula:

*κ*=(*po*−*pe)/(*1−*pe)*​​​

Among them:

po is the observed consistency,i.e.the proportion of two reviewers making the same judgement.

pe is random consistency,i.e.the expected proportion of two reviewers making the same judgement due to random chance.

*Po=22/25*

*Pe=1/3*

*κ*=0.82

Kappa values are usually interpreted as follows:

0.01-0.20:slight consistency

0.21-0.40:fair consistency

0.41-0.60:moderate consistency

0.61-0.80:good consistency

0.81-1.00:very good consistency

Our analyses have very good consistency

eFigure2.Kappa of ivermectin for Covid-19


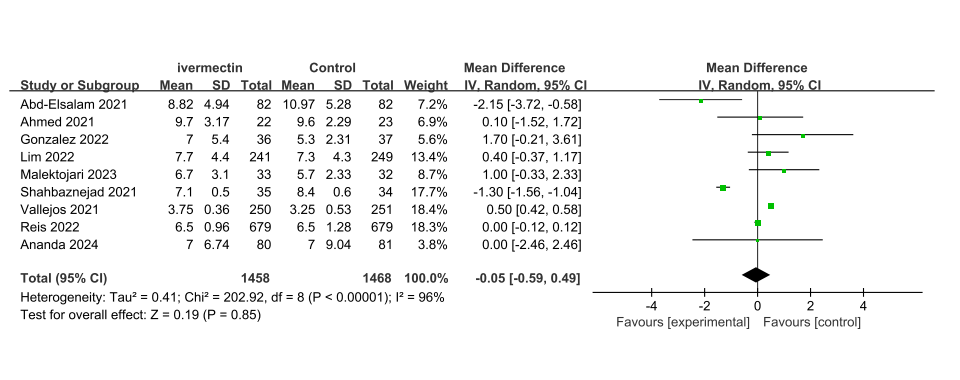


eFigure3.Ivermectin on the length of hospital stay


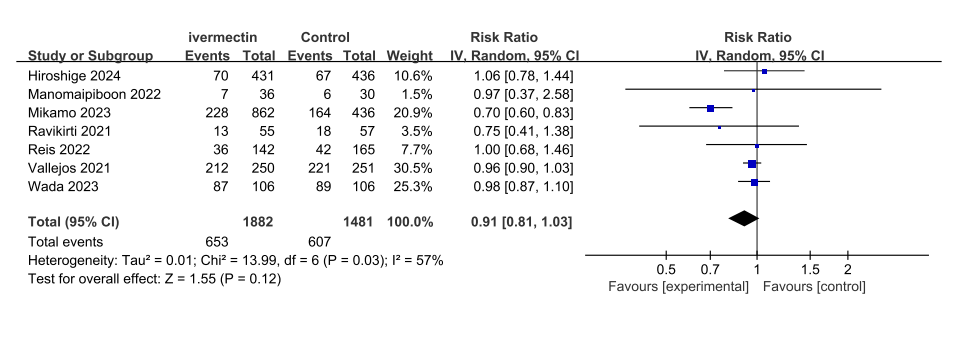
eFigure4.Rate of negative COVID-19 tests


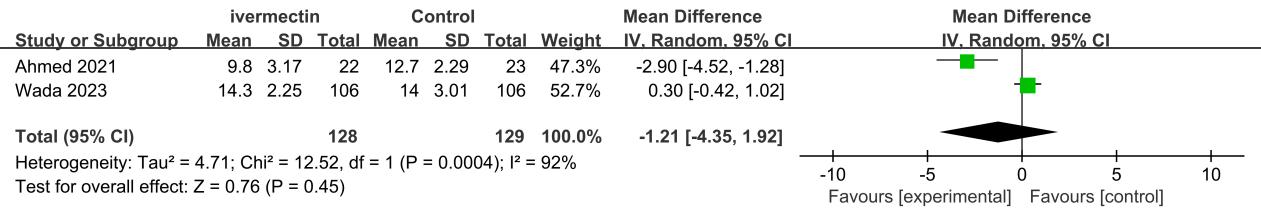
eFigure5.Mean duration to viral clearance


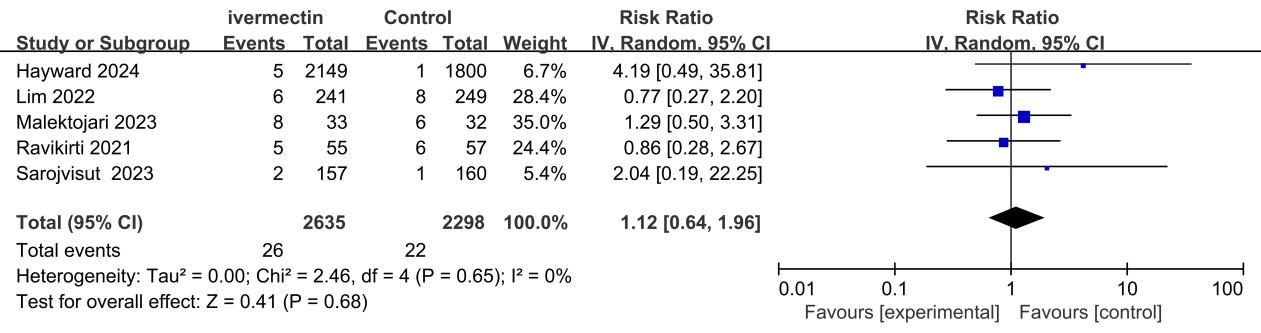
eFigure6.Rates of ICU admission


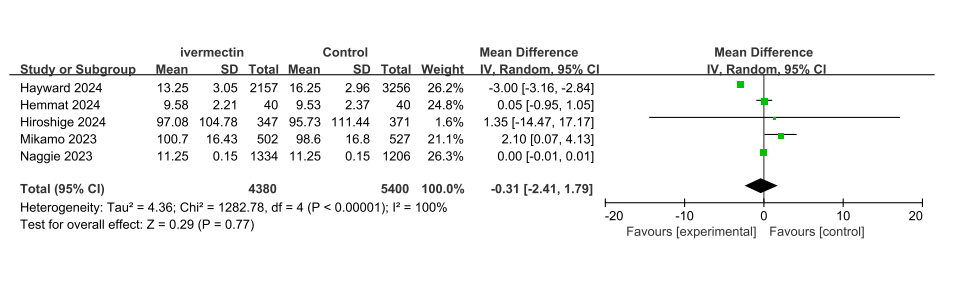
eFigure7.Recovery time


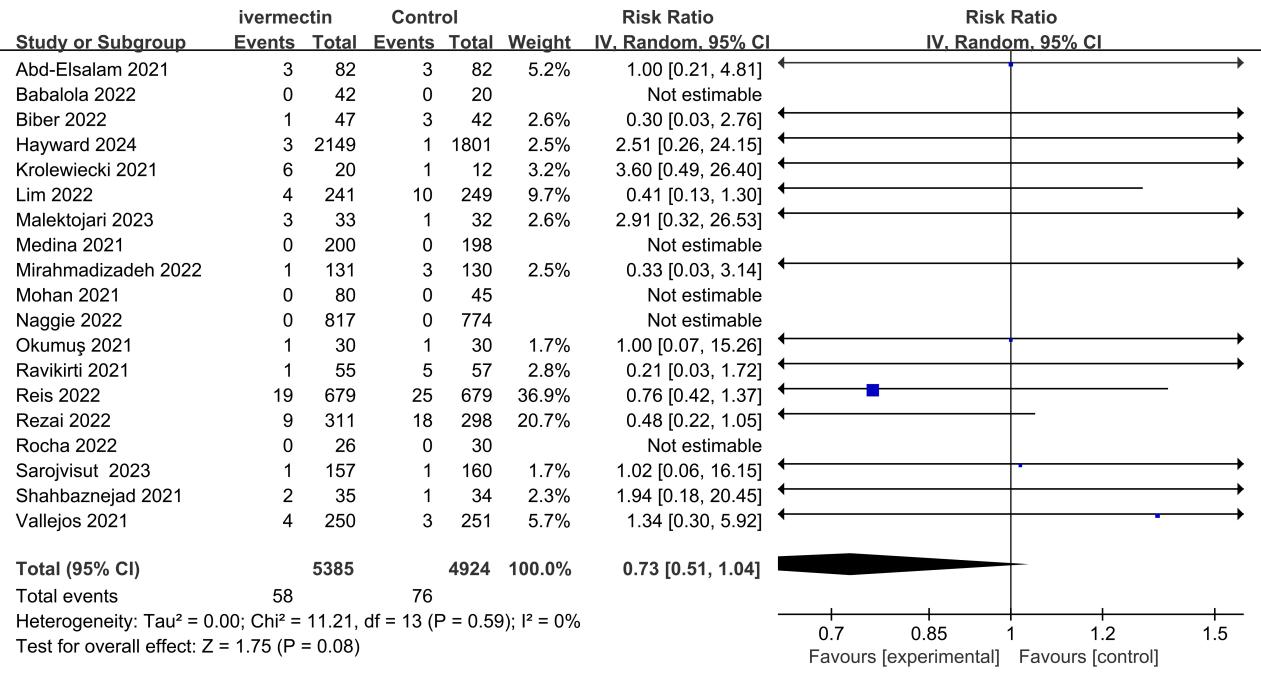
eFigure8.Rate of mechanical ventilation


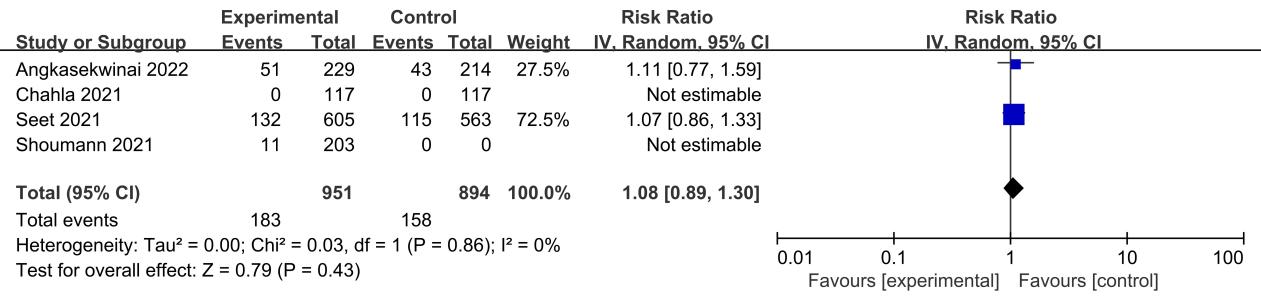
eFigure9.preventive group with adverse events


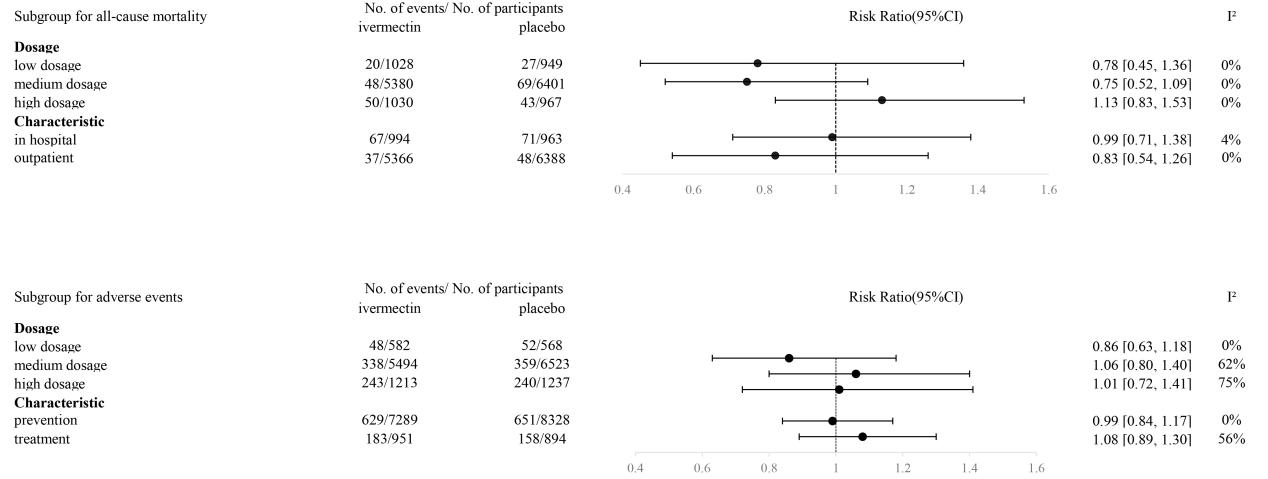


eFigure10.Subgroup analysis on patients with low dosage,medium dosage and high dosage.


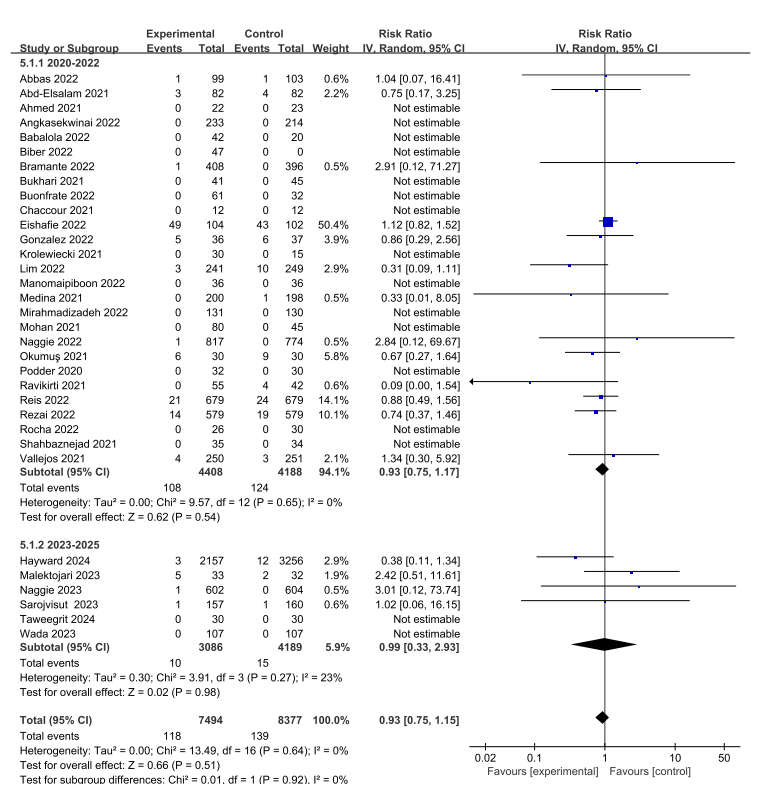
eFigure11.time-stratified subgroup on reducing all-cause mortality


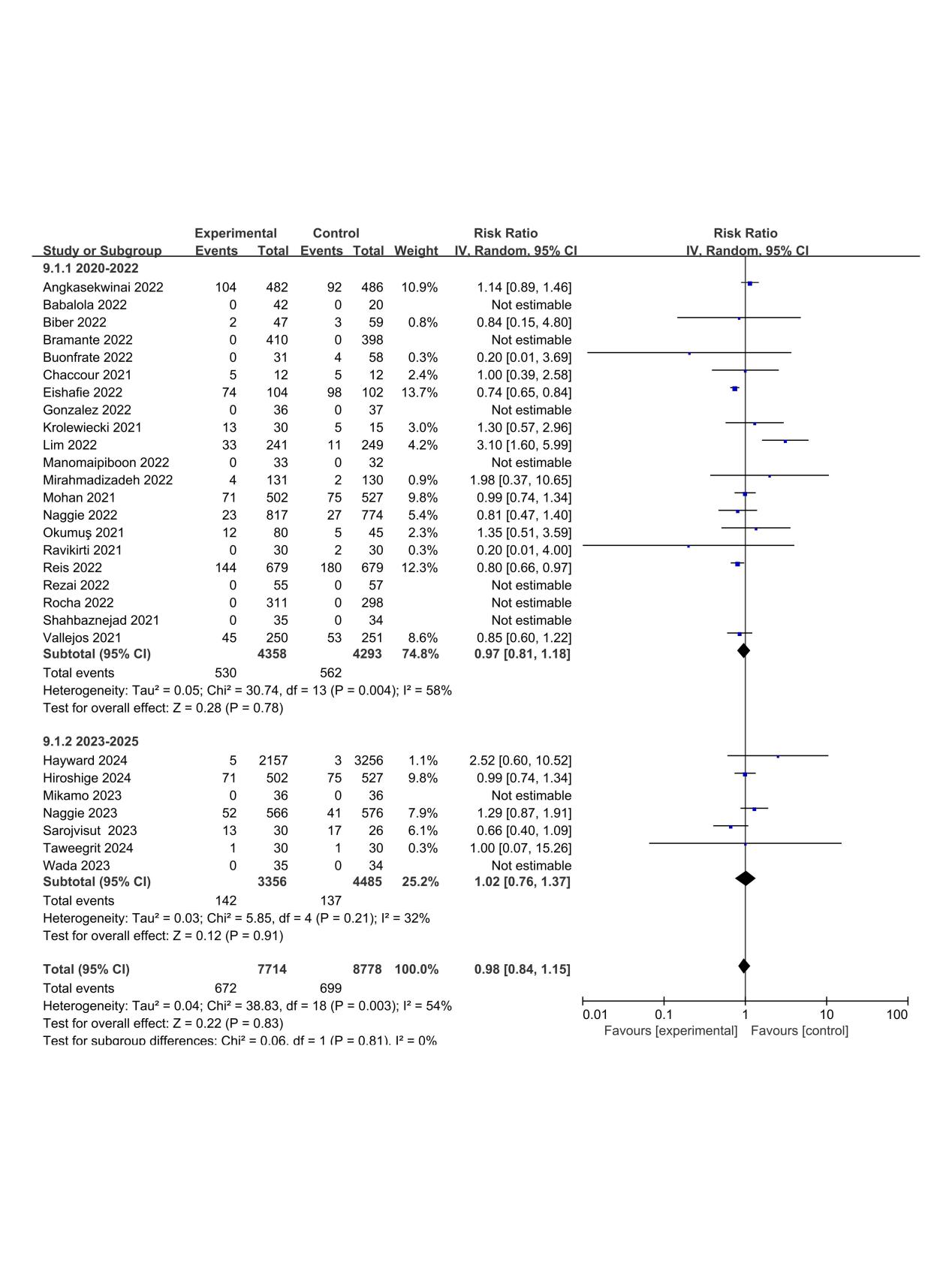
eFigure12.time-stratified subgroup on reducing adverse event


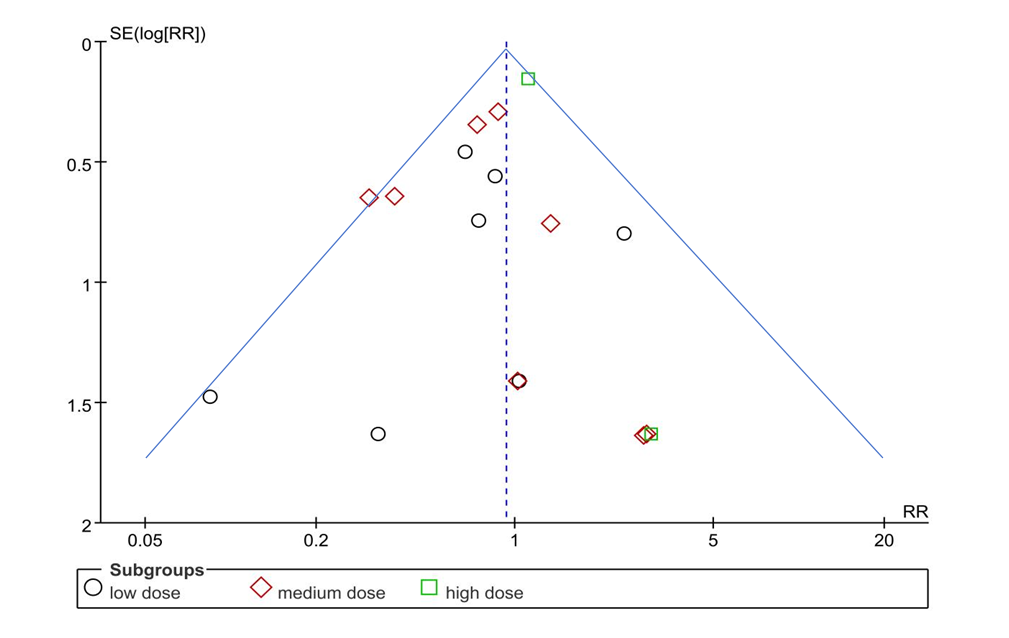


eFigure13.funnel plot of all-cause morality


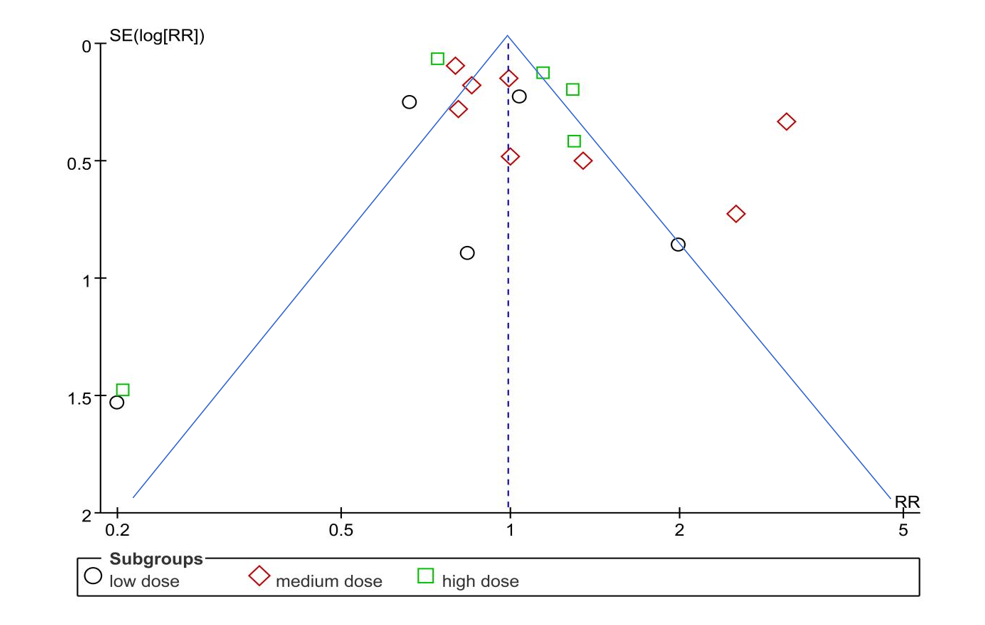


eFigure14.funnel plot of adverse events


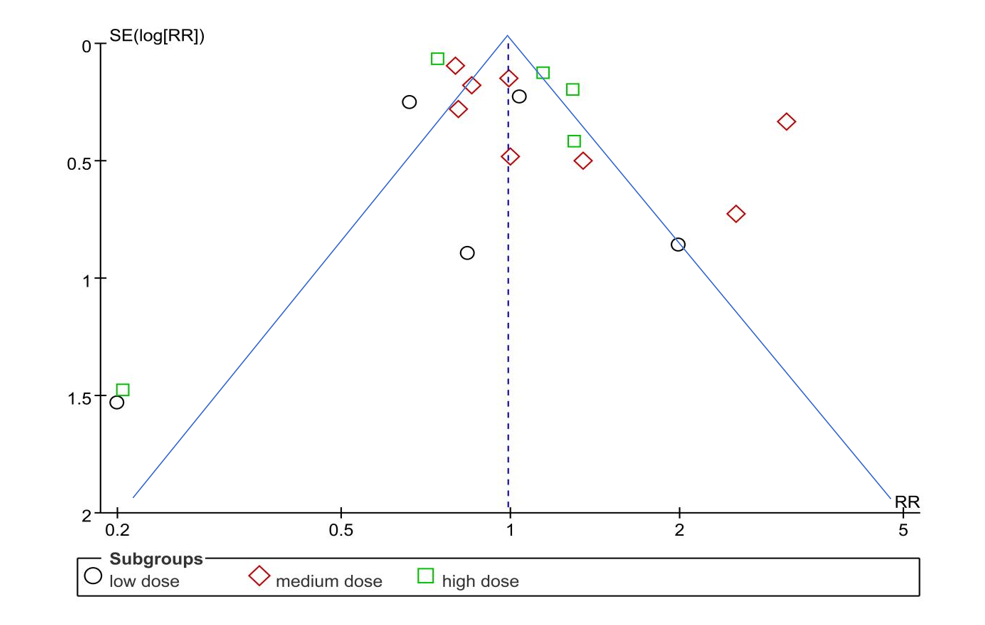

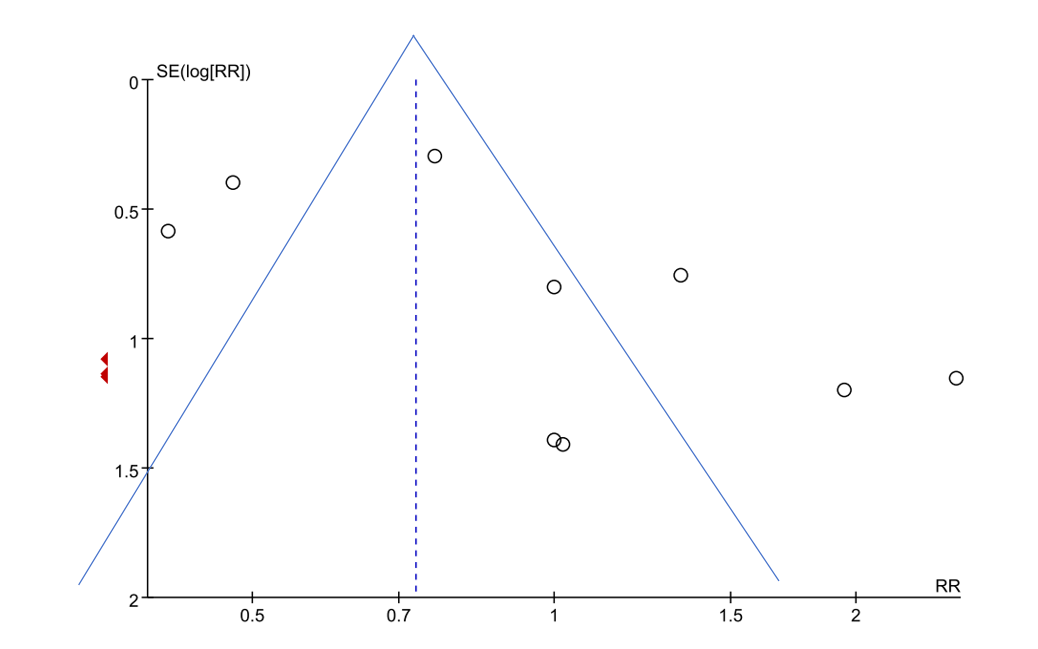


eFigure15.funnel plot of mechanical ventilation

| Population | Outcomes | Risk of bias | Inconsistency | lmprecision | Small study effects | Certainty of evidence |
| --- | --- | --- | --- | --- | --- | --- |
| Prevention | preventive effects | Downgraded | Not downgraded | Not downgraded | Not downgraded | Low |
|  | adverse events | Downgraded | Downgraded | Not downgraded | Not downgraded | Low |
| Treatment | all-cause mortality | Not Downgraded | Not Downgraded | Not Downgraded | Not Downgraded | High |
|  | adverse events | Not Downgraded | Not Downgraded | Not Downgraded | Not Downgraded | High |
|  | length of hospital stay | Downgraded | Downgraded | Downgraded | Not downgraded | Moderate |
|  | the rate of negative COVID-19 tests | Downgraded | Downgraded | Downgraded | Not downgraded | Moderate |
|  | the mean duration to viral clearance | Not downgraded | Downgraded | Not downgraded | Downgraded | Low |
|  | rates of ICU admission | Not downgraded | Downgraded | Not downgraded | Downgraded | Low |
| Treatment | recovery time | Not downgraded | Downgraded | Downgraded | Downgraded | Low |
|  | mechanical ventilation | Downgraded | Not downgraded | Downgraded | Not downgraded | Moderate |

Risk of bias:Downgraded by one level because>259 of participants in this comparison were from studies at high risk of bias.

Inconsistency lmprecision:Downgraded by one level because heterogeneity(12)>50%.

Small study:Downgraded by one level because the limits of the 95%confidence interval were 20%different to the point estimates.

Effects:Downgraded by one level owing to small study bias.

eTable 1.Grading of ivermectin for Covid-19

1.Suppose a study is available that presents the following information:

|  | Baseline | Final | Change |
| --- | --- | --- | --- |
| Experimental intervention (sample size n1) | \| meanl \| (B),SD1 \| (B) \| \| --- \| --- \| --- \| | \| meanl \| (F),SD1 \| (F) \| \| --- \| --- \| --- \| | \| meanl \| (C),SD1 \| (C) \| \| --- \| --- \| --- \| |
| Control intervention (sample size n2) | \| mean2 \| (B),SD2 \| (B) \| \| --- \| --- \| --- \| | \| mean2 \| (F),SD2 \| (F) \| \| --- \| --- \| --- \| | \| mean2 \| (C),SD2 \| (C) \| \| --- \| --- \| --- \| |

An analysis of change from baseline is available from this study,using only the data in the final column.We can use the other data from the study to estimate the correlation coefficient in the experimental intervention,rl,as follows:


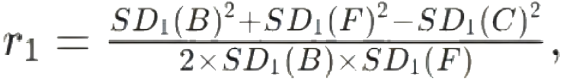


and similarly for the control intervention, r2.Where either SD(F) or SD(B)are unavailable,then it may be substituted by the other if it is reasonable to assume that the intervention does not alter the variability of the outcome measure.Correlation coefficients lie between-1 and If zero or a negative number is obtained,then there is no value in using change from baseline and an analysis of final values should be performed.Assuming thecorrelation coefficients from the two intervention groups are similar,a simple average will provide a reasonable measure of the similarity of baseline.

*SD₁(C)=√(SD₁(B)²+SD₁(F)²-(2×R₁×SD₁(B)×SD₁(F))),*

and similarly for the control intervention.Again,if either SD(F) or SD(B) are unavailable,then one may be substituted by the other if it is reasonable to assume that the intervention does not alter the variability of the outcome measure.

As an example,given the following data:

|  | \| Baseline \| Final \| \| --- \| --- \| | | Change |
| --- | --- | --- | --- | --- | --- |
| Experimental intervention (sample size 35) | mean=12.4 SD=4.2 | mean=15.2 SD=3.8 | mean=2.8 |
| Control intervention (sample size 38) | mean=10.7 SD=4.0 | mean=13.8 SD=4.4 | mean=3.1 |

and using an imputed correlation coefficient of 0.5,we can impute the standard deviation for the change score in the control group

as:SD2 (C)=4.02+4.42-(2×0.5×4.0×4.0) =4.21

eTable 2.specific calculation methodology of MD and SD

| Study | Location | Patient Characteristics | Treatment | |
| --- | --- | --- | --- | --- |
|  |  |  | Intervention | Control |
| Abbas 2022(1) | China | hospitalized patients | Ivermectin 18 mg, oral, once daily for 5 consecutive days | placebo |
| Abd-Elsalam 2021(2) | Egypt | hospitalized patients | Ivermectin 12 mg, oral, once daily for 5 consecutive days | placebo |
| Ahmed 2021(3) | Bangladesh | hospitalized patients | Ivermectin 12 mg single dose | placebo |
| Ananda 2024(4) | Colombo | hospitalized patients | Ivermectin 6 mg single dose | placebo |
| Angkasekwinai 2022(5) | Thailand | outpatient group | Ivermectin 18 mg, oral, once daily for 3 consecutive days | placebo |
| Babalola 2022(6) | Nigeria | NA | Ivermectin 6 mg twice weekly | placebo |
| Biber 2022(7) | Israel | outpatient group | Ivermectin 12 mg, oral, once daily for 3 consecutive days | placebo |
| Bramante 2022(8) | USA | outpatient group | Ivermectin 24 mg, oral, once daily for 3 consecutive days | placebo |
| Bukhari 2021(9) | Pakistan | hospitalized patients | Ivermectin 12 mg single dose | placebo |
| Buonfrate 2022(10) | Italy | outpatient group | Ivermectin 36 mg, oral, once daily for 3 consecutive days | placebo |
| Carolyn 2024(11) | multisite | outpatient group | Ivermectin 24 mg, oral, once daily for 3 consecutive days | placebo |
| Chaccour 2021(12) | Spain | outpatient group | Ivermectin 24 mg single dose | placebo |
| Chahla,2021(13) | Argentina | prevention | Ivermectin 24 mg, oral, once daily for 3 consecutive days | placebo |
| Elshafie 2022(14) | Pakistan | hospitalized patients | Ivermectin 24 mg, oral, once daily for 3 consecutive days | placebo |
| Gonzalez 2022(15) | Aguascalientes(Mexico) | hospitalized patients | Ivermectin 12 mg, oral, once daily for 3 consecutive days | placebo |
| Hayward 2024(16) | UK national | outpatient group | Ivermectin 24 mg, oral, once daily for 3 consecutive days | placebo |
| Hemmat 2024(17) | Egypt | outpatient group | Ivermectin 6 mg single dose | placebo |
| Hiroshige 2024(18) | Japan and Thailand | NA | Ivermectin 24 mg single dose | placebo |
| Krolewiecki 2021(19) | Argentina | hospitalized patients | Ivermectin 36 mg, oral, once daily for 5 consecutive days | placebo |
| lim 2022(20) | Malaysia | hospitalized patients | Ivermectin 24 mg, oral, once daily for 5 consecutive days | placebo |
| Malektojari 2023(21) | Iran | hospitalized patients | Ivermectin 12 mg single dose | placebo |
| Manomaipiboon 2022(22) | Thailand | hospitalized patients | Ivermectin 12 mg, oral, once daily for 5 consecutive days | placebo |
| Medina 2021(23) | Colombia | NA | Ivermectin 18 mg, oral, once daily for 3 consecutive days | placebo |
| Mirahmadizadeh 2022(24) | Iran | outpatient group | Ivermectin 6 mg, oral, once daily for 5 consecutive days | placebo |
| Mohan 2021(25) | India | hospitalized patients | Ivermectin 24 mg single dose | placebo |
| Naggie 2022(26) | US | outpatient group | Ivermectin 24 mg, oral, once daily for 3 consecutive days | placebo |
| Naggie 2023(27) | US | outpatient group | Ivermectin 36mg, oral, once daily for 5 consecutive days | placebo |
| Okumuş2021(28) | Turkey | hospitalized patients | Ivermectin 12mg, oral, once daily for 5 consecutive days | placebo |
| Podder 2020(29) | Bangladesh | NA | Ivermectin 12 mg single dose | placebo |
| Ravikirti 2021(30) | India | hospitalized patients | Ivermectin 12 mg single dose | placebo |
| Reis 2022(31) | Brazil | outpatient group | Ivermectin 24 mg, oral, once daily for 3 consecutive days | placebo |
| Rezai 2022(32) | Iran | NA | Ivermectin 24 mg, oral, once daily for 3 consecutive days | placebo |
| Rocha 2022(33) | Mexico | NA | Ivermectin 12 mg, oral, once daily for 3 consecutive days | placebo |
| Sarojvisut 2023(34) | Thailand | hospitalized patients | Ivermectin 24 mg, oral, once daily for 3 consecutive days | placebo |
| Seet 2021(35) | Singapore | prevention | Ivermectin 12 mg single dose | placebo |
| Shahbaznejad 2021(36) | Iran | hospitalized patients | Ivermectin 12 mg single dose | placebo |
| Shoumann 2021(37) | Egypt | NA | Ivermectin 18 mg, oral, once daily for 3 consecutive days | placebo |
| Taweegrit 2024(38) | Thailand | outpatient group | Ivermectin 24 mg, oral, once daily for 3 consecutive days | placebo |
| Vallejos 2021(39) | Argentina | outpatient group | Ivermectin 24 mg, oral, once daily for 3 consecutive days | placebo |
| Wada 2023(40) | Japan | hospitalized patients | Ivermectin 12 mg, oral, once daily for 3 consecutive days | placebo |

eTable 3.Characteristics of included RCTs

**eAppendix 1.**Reference list of included studies

1. Abbas K,Muhammad S,Ding S.2022.The Effect of Ivermectin on Reducing Viral Symptoms in Patients with Mild COVID-19.Indian Journal of Pharmaceutical Sciences 84.

2. Abd-Elsalam S,Noor RA,Badawi R,Khalaf M,Esmail ES,Soliman S,Abd El Ghafar MS,Elbahnasawy M,Moustafa EF,Hassany SM,Medhat MA,Ramadan HK,Eldeen MAS,Alboraie M,Cordie A,Esmat G.2021.Clinical study evaluating the efficacy of ivermectin in COVID-19 treatment:A randomized controlled study.J Med Virol 93:5833-5838.

3. Ahmed S,Karim MM,Ross AG,Hossain MS,Clemens JD,Sumiya MK,Phru CS,Rahman M,Zaman K,Somani J,Yasmin R,Hasnat MA,Kabir A,Aziz AB,Khan WA.2021.A five-day course of ivermectin for the treatment of COVID-19 may reduce the duration of illness.Int J Infect Dis 103:214-216.

4. Wijewickrema A,Banneheke H,Pathmeswaran A,Refai FW,Kauranaratne M,Malavige N,Jeewandara C,Ekanayake M,Samaraweera D,Thambavita D,Galappatthy P.2024.Efficacy and safety of oral ivermectin in the treatment of mild to moderate Covid-19 patients:a multi-centre double-blind randomized controlled clinical trial.BMC Infect Dis 24:719.

5. Angkasekwinai N,Rattanaumpawan P,Chayakulkeeree M,Phoompoung P,Koomanachai P,Chantarasut S,Wangchinda W,Srinonprasert V,Thamlikitkul V.2022.Safety and Efficacy of Ivermectin for the Prevention and Treatment of COVID-19:A Double-Blinded Randomized Placebo-Controlled Study.Antibiotics(Basel)11.

6. Babalola O,Bode C,Ajayi A,Alakaloko F,Akase I,Otrofanowei E,Salu O,Adeyemo WL,Ademuyiwa A,Omilabu S.2021.Ivermectin shows clinical benefits in mild to moderate COVID19:A randomised controlled double-blind,dose-response study in Lagos.QJM:An International Journal of Medicine 114.

7. Biber A,Harmelin G,Lev D,Ram L,Shaham A,Nemet I,Kliker L,Erster O,Mandelboim M,Schwartz E.2022.The effect of ivermectin on the viral load and culture viability in early treatment of non-hospitalized patients with mild COVID-19–A double-blind,randomized placebo-controlled trial.International Journal of Infectious Diseases 122.

8. Bramante CT,Huling JD,Tignanelli CJ,Buse JB,Liebovitz DM,Nicklas JM,Cohen K,Puskarich MA,Belani HK,Proper JL,Siegel LK,Klatt NR,Odde DJ,Luke DG,Anderson B,Karger AB,Ingraham NE,Hartman KM,Rao V,Hagen AA,Patel B,Fenno SL,Avula N,Reddy NV,Erickson SM,Lindberg S,Fricton R,Lee S,Zaman A,Saveraid HG,Tordsen WJ,Pullen MF,Biros M,Sherwood NE,Thompson JL,Boulware DR,Murray TA.2022.Randomized Trial of Metformin,Ivermectin,and Fluvoxamine for Covid-19.N Engl J Med 387:599-610.

9. Bukhari S,Asghar A,Perveen N,Hayat A,Mangat S,Butt K,Abdullah M,Fatima T,Mustafa A,Cheema T.2021.Efficacy of Ivermectin in COVID-19 Patients with Mild to Moderate Disease doi:10.1101/2021.02.02.21250840.

10. Buonfrate D,Chesini F,Martini D,Roncaglioni MC,Ojeda Fernandez ML,Alvisi MF,De Simone I,Rulli E,Nobili A,Casalini G,Antinori S,Gobbi M,Campoli C,Deiana M,Pomari E,Lunardi G,Tessari R,Bisoffi Z.2022.High-dose ivermectin for early treatment of COVID-19(COVER study):a randomised,double-blind,multicentre,phase II,dose-finding,proof-of-concept clinical trial.Int J Antimicrob Agents 59:106516.

11. Bramante CT,Beckman KB,Mehta T,Karger AB,Odde DJ,Tignanelli CJ,Buse JB,Johnson DM,Watson RHB,Daniel JJ,Liebovitz DM,Nicklas JM,Cohen K,Puskarich MA,Belani HK,Siegel LK,Klatt NR,Anderson B,Hartman KM,Rao V,Hagen AA,Patel B,Fenno SL,Avula N,Reddy NV,Erickson SM,Fricton RD,Lee S,Griffiths G,Pullen MF,Thompson JL,Sherwood NE,Murray TA,Rose MR,Boulware DR,Huling JD.2024.Favorable Antiviral Effect of Metformin on SARS-CoV-2 Viral Load in a Randomized,Placebo-Controlled Clinical Trial of COVID-19.Clin Infect Dis 79:354-363.

12. Chaccour C,Casellas A,Blanco A,PinedaÍ,Fernandez-Montero A,Ruiz-Castillo P,Richardson M-A,Rodríguez-Mateos M,Jordán-Iborra C,Brew J,Carmona-Torre FdA,Giraldez M,Laso E,Gabaldón-Figueira J,Dobaño Lázaro C,Moncunill G,Yuste J,del Pozo J,Rabinovich N,Fernández-Alonso M.2021.The effect of early treatment with ivermectin on viral load,symptoms and humoral response in patients with non-severe COVID-19:A pilot,double-blind,placebo-controlled,randomized clinical trial.EClinicalMedicine 32:100720.

13. Chahla RE,Medina Ruiz L,Ortega ES,Morales Rn MF,Barreiro F,George A,Mancilla Rn C,S DAR,Barrenechea G,Goroso DG,Peral de Bruno M.2021.Intensive Treatment With Ivermectin and Iota-Carrageenan as Pre-exposure Prophylaxis for COVID-19 in Health Care Workers From Tucuman,Argentina.Am J Ther 28:e601-e604.

14. Elshafie A,Elsawah H,Hammad M,Sweed E,Seif A,Mostafa M,Goda F,Mosalam E,Abdallah M.2022.Ivermectin Role in COVID-19 Treatment(IRICT):single center,adaptive,randomized,double-blind,placebo controlled,clinical trial.Expert review of anti-infective therapy 20.

15. Beltran Gonzalez JL,González Gámez M,Mendoza Enciso EA,Esparza Maldonado RJ,Hernández Palacios D,Dueñas Campos S,Robles IO,Macías Guzmán MJ,García Díaz AL,Gutiérrez Peña CM,Martinez Medina L,Monroy Colin VA,Arreola Guerra JM.2022.Efficacy and Safety of Ivermectin and Hydroxychloroquine in Patients with Severe COVID-19:A Randomized Controlled Trial.Infect Dis Rep 14:160-168.

16. Hayward G,Yu LM,Little P,Gbinigie O,Shanyinde M,Harris V,Dorward J,Saville BR,Berry N,Evans PH,Thomas NPB,Patel MG,Richards D,Hecke OV,Detry MA,Saunders C,Fitzgerald M,Robinson J,Latimer-Bell C,Allen J,Ogburn E,Grabey J,de Lusignan S,Hobbs FR,Butler CC.2024.Ivermectin for COVID-19 in adults in the community(PRINCIPLE):An open,randomised,controlled,adaptive platform trial of short-and longer-term outcomes.J Infect 88:106130.

17. Ahmed Salama HAE,Ahmed EE,Amin GEE,Allam MF,Hassan ANE,Hassan El Shayeb M.2024.Role of ivermectin and colchicine in the treatment of COVID-19:a randomized controlled clinical trial.J Infect Dev Ctries 18:S298-s304.

18. Mikamo H,Takahashi S,Yamagishi Y,Hirakawa A,Harada T,Nagashima H,Noguchi C,Masuko K,Maekawa H,Kashii T,Ohbayashi H,Hosokawa S,Maejima K,Yamato M,Manosuthi W,Paiboonpol S,Suganami H,Tanigawa R,Kawamura H.2024.Efficacy and safety of ivermectin in patients with mild COVID-19 in Japan and Thailand.J Infect Chemother 30:536-543.

19. Krolewiecki A,Lifschitz A,Moragas M,Travacio M,Valentini R,Alonso D,Solari R,Tinelli M,Cimino R,Alvarez L,Fleitas P,Ceballos L,Golemba M,Fernández F,Oliveira D,Astudillo G,Baeck I,Farina J,Cardama G,Lanusse C.2021.Antiviral effect of high-dose ivermectin in adults with COVID-19:A proof-of-concept randomized trial.EClinicalMedicine 37:100959.

20. Lim SCL,Hor CP,Tay KH,Mat Jelani A,Tan WH,Ker HB,Chow TS,Zaid M,Cheah WK,Lim HH,Khalid KE,Cheng JT,Mohd Unit H,An N,Nasruddin AB,Low LL,Khoo SWR,Loh JH,Zaidan NZ,Ab Wahab S,Song LH,Koh HM,King TL,Lai NM,Chidambaram SK,Peariasamy KM.2022.Efficacy of Ivermectin Treatment on Disease Progression Among Adults With Mild to Moderate COVID-19 and Comorbidities:The I-TECH Randomized Clinical Trial.JAMA Intern Med 182:426-435.

21. Malektojari A,Ghazizadeh S,Ersi MH,Brahimi E,Hassanipour S,Fathalipour M,Hassaniazad M.2023.Efficacy and safety of ivermectin in patients with mild and moderate COVID-19:A randomized controlled trial.16:3-8.

22. Manomaipiboon A,Pholtawornkulchai K,Poopipatpab S,Suraamornkul S,Maneerit J,Ruksakul W,Phumisantiphong U,Trakarnvanich T.2022.Efficacy and safety of ivermectin in the treatment of mild to moderate COVID-19 infection:a randomized,double-blind,placebo-controlled trial.Trials 23:714.

23. Lopez-Medina E,Lopez P,Hurtado I,Dávalos D,Ramirez O,Martinez E,Díazgranados J,Oñate J,Chavarriaga H,Herrera S,Parra B,Libreros G,Jaramillo R,Avendaño A,Toro D,Torres M,Lesmes M,Rios C,Caicedo I.2021.Effect of Ivermectin on Time to Resolution of Symptoms among Adults with Mild COVID-19:A Randomized Clinical Trial.JAMA 325.

24. Mirahmadizadeh A,Semati A,Heiran A,Ebrahimi M,Hemmati A,Basir S,Zare M,Da-Costa A,Zeinali M,Sargolzaee M,Eilami O.2022.Efficacy of single‐dose and double‐dose ivermectin early treatment in preventing progression to hospitalization in mild COVID‐19:A multi‐arm,parallel‐group randomized,double‐blind,placebo‐controlled trial.Respirology 27.

25. Mohan A,Tiwari P,Suri T,Mittal S,Patel A,Jain A,Thirumurthy V,Das U,Boppana T,Pandey R,Shelke S,Singh A,Bhatnagar S,Masih S,Mahajan S,Dwivedi T,Sahoo B,Pandit A,Bhopale S,Guleria R.2021.Single-dose oral ivermectin in mild and moderate COVID-19(RIVET-COV):A single-centre randomized,placebo-controlled trial.Journal of Infection and Chemotherapy 27.

26. Naggie S.2022.Ivermectin for Treatment of Mild-to-Moderate COVID-19 in the Outpatient Setting:A Decentralized,Placebo-controlled,Randomized,Platform Clinical Trial.medRxiv doi:10.1101/2022.06.10.22276252.

27. Naggie S,Boulware DR,Lindsell CJ,Stewart TG,Slandzicki AJ,Lim SC,Cohen J,Kavtaradze D,Amon AP,Gabriel A,Gentile N,Felker GM,Jayaweera D,McCarthy MW,Sulkowski M,Rothman RL,Wilson S,DeLong A,Remaly A,Wilder R,Collins S,Dunsmore SE,Adam SJ,Thicklin F,Hanna GJ,Ginde AA,Castro M,McTigue K,Shenkman E,Hernandez AF.2023.Effect of Higher-Dose Ivermectin for 6 Days vs Placebo on Time to Sustained Recovery in Outpatients With COVID-19:A Randomized Clinical Trial.Jama 329:888-897.

28. OkumuşN,Demirturk N,AytaçC,Guner R,AvciİY,Orhan S,Konya P,Şaylan B,Karalezli A,Yamanel L,Kayaaslan B,Yılmaz G,SavaşçıÜ,Eser F,Taşkın G.2021.Evaluation of the effectiveness and safety of adding ivermectin to treatment in severe COVID-19 patients.BMC Infectious Diseases 21.

29. Podder C,Chowdhury N,Sina M,Haque W.2020.Outcome of ivermectin treated mild to moderate COVID-19 cases:a single-centre,open-label,randomised controlled study.IMC Journal of Medical Science 14.

30. Ravikirti,Roy R,Pattadar C,Raj R,Agarwal N,Biswas B,Manjhi PK,Rai DK,Shyama,Kumar A,Sarfaraz A.2021.Evaluation of Ivermectin as a Potential Treatment for Mild to Moderate COVID-19:A Double-Blind Randomized Placebo Controlled Trial in Eastern India.J Pharm Pharm Sci 24:343-350.

31. Reis G,Silva E,Silva DCM,Thabane L,Milagres AC,Ferreira TS,Dos Santos CVQ,Campos VHS,Nogueira AMR,de Almeida A,Callegari ED,Neto ADF,Savassi LCM,Simplicio MIC,Ribeiro LB,Oliveira R,Harari O,Forrest JI,Ruton H,Sprague S,McKay P,Guo CM,Rowland-Yeo K,Guyatt GH,Boulware DR,Rayner CR,Mills EJ.2022.Effect of Early Treatment with Ivermectin among Patients with Covid-19.N Engl J Med 386:1721-1731.

32. Rezai MS,Ahangarkani F,Hill A,Ellis L,Mirchandani M,Davoudi A,Eslami G,Roozbeh F,Babamahmoodi F,Rouhani N,Alikhani A,Najafi N,Ghasemian R,Mehravaran H,Hajialibeig A,Navaifar M,Shahbaznejad L,Rahimzadeh G,Saeedi M,Valadan R.2022.Non-effectiveness of Ivermectin on Inpatients and Outpatients With COVID-19;Results of Two Randomized,Double-Blinded,Placebo-Controlled Clinical Trials.Frontiers in Medicine 9:919708.

33. de la Rocha C,Cid-López MA,Venegas-López BI,Gómez-Méndez SC,Sánchez-Ortiz A,Pérez-Ríos AM,Llamas-Velázquez RA,Meza-Acuña AI,Vargas-Íñiguez B,Rosales-Galván D,Tavares-Váldez A,Luna-Gudiño N,Hernández-Puente CV,Milenkovic J,Iglesias-Palomares C,Méndez-Del Villar M,Gutiérrez-Dieck GA,Valderrábano-Roldán CG,Mercado-Cerda J,Robles-Bojórquez JG,Mercado-Sesma AR.2022.Ivermectin compared with placebo in the clinical course in Mexican patients with asymptomatic and mild COVID-19:a randomized clinical trial.BMC Infect Dis 22:917.

34. Sarojvisut P,Apisarnthanarak A,Jantarathaneewat K,Sathitakorn O,Pienthong T,Mingmalairak C,Warren DK,Weber DJ.2023.An Open Label Randomized Controlled Trial of Ivermectin Plus Favipiravir-Based Standard of Care versus Favipiravir-Based Standard of Care for Treatment of Moderate COVID-19 in Thailand.Infect Chemother 55:50-58.

35. Seet RCS,Quek AML,Ooi DSQ,Sengupta S,Lakshminarasappa SR,Koo CY,So JBY,Goh BC,Loh KS,Fisher D,Teoh HL,Sun J,Cook AR,Tambyah PA,Hartman M.2021.Positive impact of oral hydroxychloroquine and povidone-iodine throat spray for COVID-19 prophylaxis:An open-label randomized trial.Int J Infect Dis 106:314-322.

36. Shahbaznejad L,Davoudi A,Eslami G,Markowitz JS,Navaeifar MR,Hosseinzadeh F,Movahedi FS,Rezai MS.2021.Effects of Ivermectin in Patients With COVID-19:A Multicenter,Double-blind,Randomized,Controlled Clinical Trial.Clin Ther 43:1007-1019.

37. Shouman W,Hegazy A,Nafae R,Sileem A.2021.Use of Ivermectin as a potential chemoprophylaxis for COVID-19 in Egypt:A Randomised clinical trial.Journal of Clinical and Diagnostic Research doi:10.7860/JCDR/2020/46795.0000.

38. Siripongboonsitti T,Tawinprai K,Avirutnan P,Jitobaom K,Auewarakul P.2024.A randomized trial to assess the acceleration of viral clearance by the combination Favipiravir/Ivermectin/Niclosamide in mild-to-moderate COVID-19 adult patients(FINCOV).J Infect Public Health 17:897-905.

39. Vallejos J,Zoni R,Bangher M,Villamandos S,Bobadilla A,Plano F,Campias C,Chaparro Campias E,Medina MF,Achinelli F,Guglielmone HA,Ojeda J,Farizano Salazar D,Andino G,Kawerin P,Dellamea S,Aquino AC,Flores V,Martemucci CN,Martinez SM,Segovia JE,Reynoso PI,Sosa NC,Robledo ME,Guarrochena JM,Vernengo MM,Ruiz Diaz N,Meza E,Aguirre MG.2021.Ivermectin to prevent hospitalizations in patients with COVID-19(IVERCOR-COVID19)a randomized,double-blind,placebo-controlled trial.BMC Infect Dis 21:635.

40. Wada T,Hibino M,Aono H,Kyoda S,Iwadate Y,Shishido E,Ikeda K,Kinoshita N,Matsuda Y,Otani S,Kameda R,Matoba K,Nonaka M,Maeda M,Kumagai Y,Ako J,Shichiri M,Naoki K,Katagiri M,Takaso M,Iwamura M,Katayama K,Miyatsuka T,Orihashi Y,Yamaoka K.2023.Efficacy and safety of single-dose ivermectin in mild-to-moderate COVID-19:the double-blind,randomized,placebo-controlled CORVETTE-01 trial.Front Med(Lausanne)10:1139046.
